# Higher Dose Anticoagulation Cannot Prevent Disease Progression in COVID-19 Patients: A Systematic Review and Meta-Analysis

**DOI:** 10.3390/biomedicines10092194

**Published:** 2022-09-05

**Authors:** Emőke Henrietta Kovács, Krisztián Tánczos, László Szabó, Caner Turan, Fanni Dembrovszky, Klementina Ocskay, Bo-Young Lee, Péter Hegyi, Zsolt Molnár

**Affiliations:** 1 Centre for Translational Medicine, Semmelweis University, 1085 Budapest, Hungary; 2 Department of Anesthesiology and Intensive Therapy, Semmelweis University, 1085 Budapest, Hungary; 3 Selye János Doctoral College for Advanced Studies, Semmelweis University, 1085 Budapest, Hungary; 4 Institute for Translational Medicine, Medical School, University of Pécs, 7624 Pécs, Hungary; 5 Division of Pancreatic Diseases, Heart and Vascular Center, Semmelweis University, 1085 Budapest, Hungary; 6 Department of Anaesthesiology and Intensive Therapy, Faculty of Medicine, Poznan University of Medical Sciences, 61-701 Poznan, Poland

**Keywords:** COVID-19, anticoagulation, thromboinflamation, disease progression

## Abstract

Implementation of higher dose (HD) thromboprophylaxis has been considered in patients infected with coronavirus disease 2019 (COVID-19). Our aim was to compare HD to standard dose (SD) thromboprophylaxis in COVID-19 patients. The protocol is registered on PROSPERO (CRD42021284808). We searched for randomised controlled studies (CENTRAL, Embase, Medline and medRxviv) that compared HD to SD anticoagulation in COVID-19 and analysed outcomes such as mortality, thrombotic events, bleedings, and disease progression. The statistical analyses were made using the random effects model. Fourteen articles were included (6253 patients). HD compared with SD showed no difference in mortality (OR 0.83 [95% CI 0.54–1.28]). The use of HD was associated with a decreased risk of thrombosis (OR 0.58 [95% CI 0.44–0.76]), although with an increased risk of major bleeding (OR 1.64 [95% CI 1.25–2.16]). The cohort with D-dimer < 1 mg/mL showed no effect (OR 1.19 [95% CI 0.67–2.11]), but in the case of D-dimer > 1 mg/mL, a tendency of lower risk in the HD group was observed (OR 0.56 [95% CI 0.31–1.00]). The need for intubation in moderately ill patients showed a nonsignificant lower likelihood in the HD group (OR 0.82 [95% CI 0.63–1.08]). We cannot advocate for HD in all COVID-19 patients, although it shows some nonsignificant benefits on disease progression in those with elevated D-dimer who do not need ICU admission.

## 1. Introduction

In December 2019, the news reported the emergence of atypical pneumonia in Wuhan, China, for the first time [1]. To date, there have been more than 6 million deaths attributed to this new virus, called severe acute respiratory syndrome coronavirus 2 (SARS-CoV2) [2].

The emergence of the SARS-CoV2 virus behind coronavirus disease 2019 (COVID-19) underlined the importance of thromboinflammatory processes after an increased number of thrombotic complications was reported and autopsies described microthrombi and fibrin deposits among their findings [3,4,5,6]. Reports have shown that coagulation parameters, namely D-dimer levels, correlate with the outcomes of COVID-19 patients [7,8].

These findings encouraged clinicians to suggest the implementation of higher dose thromboprophylaxis in the case of COVID-19 patients, typically with low molecular weight (LMWH) and unfractionated heparin (UFH) [9,10]. Besides their anticoagulant benefits, they show antiinflammatory properties and might improve clinical outcomes [11]. Thus, questions arose concerning the optimal dose of anticoagulation in COVID-19. The administration of intermediate or therapeutic dose anticoagulation has resulted in somewhat controversial findings based on observational studies [12,13,14,15,16,17,18].

Thus, there was an urgent call for randomized controlled trials (RCTs) on this topic. The HEP-COVID [19] trial showed significant benefits of survival in the case of moderately ill patients, but not in those admitted to ICU when they were administered therapeutic dose anticoagulation compared with usual-care thromboprophylaxis. The multiplatform trials by REMAP-cap, ATTAC, and ACTIV-4b investigators found that therapeutic dose thromboprophylaxis was associated with an increased number of organ-support-free days in moderately ill patients, which was further confirmed by the results of the RAPID trial [20,21]. Other trials with similar patient populations failed to show any significant advantage of the higher dose thromboprophylaxis in COVID-19 patients [22,23,24,25].

As new findings are published, there is a need for a newly updated synthesis that could shed new light on the topic and determine more precisely the patient population who would and those who would not benefit from higher dose anticoagulation.

The aim of this systematic review is to assess whether higher dose (HD) compared with standard dose (SD) versions of thromboprophylaxis have different effects on clinical outcomes without jeopardizing the safety of COVID-19 patients.

## 2. Materials and Methods

### 2.1. Research Question

We performed a meta-analysis in accordance with the Cochrane Handbook for Systematic Reviews of Interventions [26]. This protocol was registered in PROSPERO, the International Database of Prospectively Registered Systematic Reviews, with the identification number CRD42021284808 (https://www.crd.york.ac.uk/prospero (accessed on 30 July 2022)) [27].

To address our research question, we included RCTs that defined their population as adults with clinically or laboratory-confirmed COVID-19 infection and that compared higher dose to standard dose thromboprophylaxis. We defined our primary outcomes as organ support-free days (defined as days without respiratory, inotrope/vasopressor support); length of hospital stay (in days); mortality (ICU mortality, in-hospital mortality, and 30-day mortality); safety outcomes such as the incidence of thrombotic events (number of arterial and venous thrombotic events); bleeding event rate (number of major bleeding events, clinically relevant non-major bleeding, and minor bleeding as per International Society on Thrombosis and Haemostasis [ISTH]); and requirement for transfusion (packed red blood cell, platelet, fresh frozen plasma, cryoprecipitate, and prothrombin complex concentrate). Our predefined secondary outcomes were as follows: change in PaO2/FiO_2_ ratio from baseline to 7 and 14 days (in mmHg); duration of supplemental O_2_ therapy (days); duration of invasive mechanical ventilation (days); duration of vasopressor/inotrope support (days); duration of renal replacement therapy (days); other adverse events (e.g., heparin-induced thrombocytopenia); progression of disease (number of patients who needed intensive care unit (ICU) admission); and variation in markers of inflammation and coagulation (e.g., D-dimer).

Because of the quick pandemic response, and thus rapidly changing guidelines and shifts in the interest in research, we adapted our analysis to assess available data. We included the number of patients that required intubation instead of the duration of mechanical ventilation, as well as progression to acute respiratory distress syndrome (ARDS).

Additionally, we included an exploratory composite outcome in our posthoc analysis that consisted of death, pulmonary embolism (PE), and the need for invasive mechanical ventilation to assess the severity of pulmonary involvement.

### 2.2. Search Strategy, Selection Process, and Data Extraction

We conducted our systematic search on 18 October 2021 and performed an updated search on 23 May 2022 in MEDLINE, Embase, and CENTRAL using the following searchkeys: “(covid* OR SARS-CoV* OR ncov OR novel coronavirus OR COVID-19 OR coronavirus) AND (thrombosis prevention OR thromboprophylaxis OR thromboembolism prophylaxis OR anticoag* OR anticoagulation OR heparin OR UFH OR LMWH OR low molecular weight heparin OR dalteparin OR tinzaparin OR enoxaparin OR clexane OR fondaparinux OR argatroban OR bivalirudin OR rivaroxaban OR apixaban OR dabigatran OR pradaxa OR edoxaban OR betrixaban)”. In addition to the databases mentioned in the protocol registered in PROSPERO, we searched an archive (medRxviv) to include the latest, although not peer-reviewed, articles published from 1 January to 23 May 2022.

Records were screened based on title, abstract, and full-text by two independent review authors (E.H.K. and C.T.), using a reference manager software. Cohen’s kappa was calculated after each step of the selection process to measure inter-rater reliability. An independent third investigator (Z.M.) resolved the disagreements.

We extracted data in a standardized data extraction sheet. In addition to the abovementioned outcomes, we retrieved the following data from the eligible articles: title, first author, year of publication, countries, study design, eligibility criteria, anticoagulant regimen, patient demographics, and interventions. Two independent review authors (E.H.K. and B.Y.L.) extracted data using the standardized data collection form, and a third independent reviewer (F.D.) resolved the disagreements.

### 2.3. Subgroup Analyses

We planned to perform subgroup analyses to reduce the heterogeneity of the pooled data according to the severity of the disease, different dosing regimens, and baseline coagulation disorders assessed by the D-dimer level.

In order to evaluate the effect of HD compared with SD in cohorts with different disease severity, we defined them according to the level of care they needed. Thus, a “severe disease cohort” was described as patients who needed ICU level care and a “moderate disease cohort” as those who did not require organ-support and thus admission to the ICU.

We defined SD as low dose, preventive thromboprophylaxis. In the HD group, we defined intermediate and therapeutic dose anticoagulation according to the guidelines of the American Society of Hematology 2021 [28]. Thus, we considered the therapeutic dose the equivalent dose of enoxaparin 1.5 mg/kg once daily or 1 mg/kg twice daily for patients with CrCl > 30 mL/min and BMI < 40 kg/m^2^, and unfractionated heparin to target aPTT in the therapeutic range as per local guidelines or anti-Xa activity 0.3–0.7 IU/mL. The intermediate dose was defined as the equivalent of enoxaparin 0.5 mg/kg once daily or 40 mg (4000 U) twice daily for patients with CrCl > 30 mL/min and BMI < 40 kg/m^2^.

The value of 1 mg/mL D-dimer level was chosen as a cut-off point as early reports showed that, above this value, at baseline, there is an 18-fold increased risk of mortality [8,29,30,31,32]. As most of the studies reported median baseline levels, we distinguished the two cohorts: studies that enrolled patients who were admitted to hospital in more than 50% of cases with D-dimer levels > 1 mg/mL and those with baseline D-dimer < 1 mg/mL.

### 2.4. Risk of Bias and Evidence Level

The risk of bias assessment was performed by two independent review authors (E.H.K. and C.T.) following the recommendations of the Cochrane Collaboration [33]. Disagreements were resolved by a third review author (F.D.). RoB2 (risk of bias assessment) tool [34] was used to assess the transparency of the included randomised controlled studies and GRADE-Pro [35] to grade the quality of evidence. Publication bias was assessed by visual inspection of the funnel plots.

### 2.5. Statistical Analysis

The statistical analyses were carried out by R (R Core Team 2021, v 4 1.1, R Foundation for Statistical Computing, Vienna, Austria) [36], using the meta (Schwarzer 2022, v5.2.0) [37] and dmetar (Cuijpers, Furukawa, and Ebert 2022, v0.0.9000) [38] packages for calculations and plots.

To assess the effect measure, we used the odds ratio (OR) with 95% confidence intervals (95% CI) for dichotomous outcomes. For this calculation, we extracted the total number of patients in each group and those with the event of interest from each study [39,40,41].

The random effects model was used for meta-analyses. The Hartung–Knapp adjustment was used in the case where the study number for the given outcome was over five [42,43]. For the pooled results, the exact Mantel–Haenszel method (without continuity correction) was applied to handle zero cell counts [44,45]. To estimate τ^2^, we used the Paule–Mandel method [46], and the Q profile method was used for calculating the confidence interval of τ^2^ [47]. Means of the Cochrane Q test and the I^2^ values were used for the assessment of statistical heterogeneity, where *p* < 0.1 was considered as statistically significant [48].

The publication bias was evaluated by a funnel plot of the logarithm of effect size and comparison with the standard error for each trial.

Outlier and influence analyses were carried out following the recommendations of Harrer et al. (2021) and Viechtbauer and Cheung (2010) [47,49].

## 3. Results

### 3.1. Systematic Search and Selection

**Figure 1:**
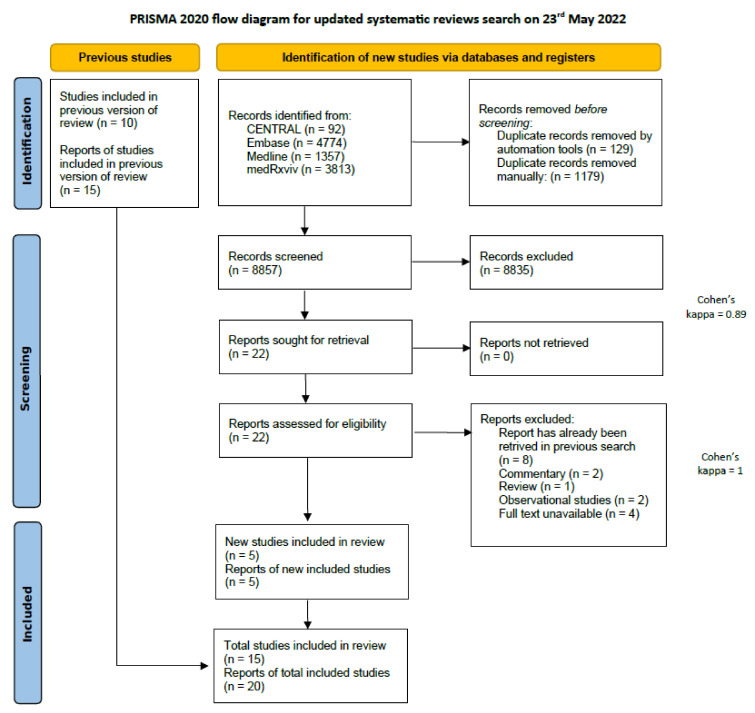
PRISMA 2020 flow diagram of the updated search.

Our systematic search resulted in 17,114 eligible studies (8749 from the search on 18 October 2021 and 8365 from 23 May 2022). After the selection process, 14 articles were included in the meta-analysis [19,20,21,22,23,24,25,50,51,52,53,54,55,56] and 15 [57] articles in the systematic review. Figure 1 shows the PRISMA 2020 Flow diagram of the updated search, and the one for the initial search can be found in Supplementary material (Figure S1) [58].

### 3.2. Study Characteristics

The included open-label studies were conducted between April 2020 and September 2021 in 15 countries [19,20,21,22,23,24,25,50,51,52,53,54,55,56]. In total, they enrolled 6253 adult patients with an average age above 50 years in the cohorts. Seven studies included patients with severe disease [50,51,52,53,54,55,56], seven with moderate disease at presentation, and one with mild disease [19,20,21,22,23,24,25,57]. The eligibility criteria of trials included higher than the upper limit of normal D-dimer levels or DIC score ≥3 in ten of the included studies [19,21,22,24,25,50,51,52,53,54,56]. Four studies compared intermediate dose thromboprophylaxis to standard dose [23,52,55]; among them, one was a three-arm study that included therapeutic anticoagulation as well [25]. The rest of the studies compared therapeutic dose to usual care thromboprophylaxis, mostly using enoxaparin [19,20,21,22,23,50,51,53,54,56]. One trial compared high dose Rivaroxaban to standard of care LMWH [22]. In a three-arm intervention study, pnyk et al. compared therapeutic UFH with therapeutic and standard dose LMWH [54]. One trial assessed the efficacy of different dosing regimens of bemiparin, while another study used tinzaparin for the same aim [24,25]. 

The characteristics of each included study can be found in Table 1. The detailed inclusion and exclusion criteria of included trials with baseline D-dimer levels can be found in Supplementary material (Table S1).

### 3.3. All-Cause Mortality

To evaluate the effectiveness of HD compared with SD anticoagulation, we extracted data about all-cause mortality reported between 21 and 30 days in 12 of the included RCTs [19,21,22,23,24,25,50,52,53,54,55,56]. This primary outcome occurred in 232 out of 1525 patients assigned to the HD group and 235 out of 1405 patients assigned to the SD group, which indicated no significant difference (OR 0.83, 95% CI [0.54–1.28], *p* = 0.3748; I^2^ = 41%, *p* = 0.06].

#### 3.3.1. Different Dosing Regimens in Moderate and Severe Disease

**Figure 2:**
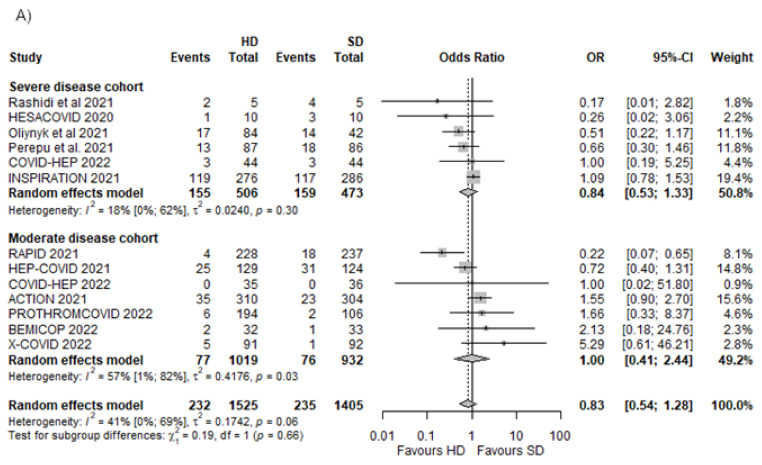

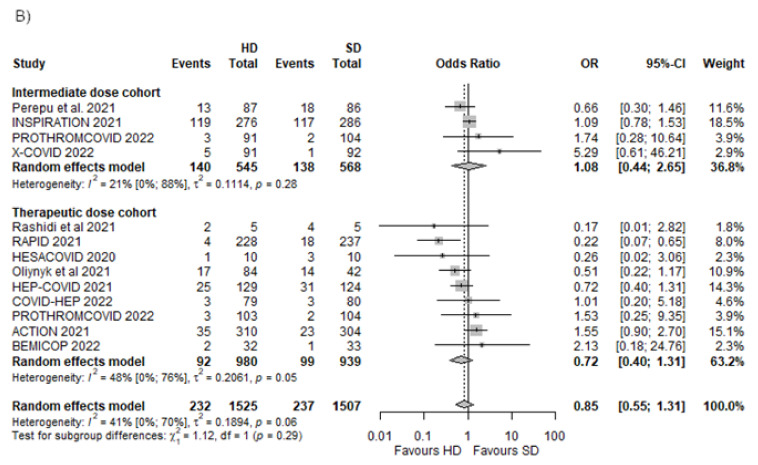
All-cause mortality in different dosing regimens in moderate and severe disease cohorts [19,21,22,23,24,25,50,52,53,54,55,56]. (**A**) All-cause mortality in severe and moderate disease cohorts; (**B**) all-cause mortality in cohorts where intermediate or therapeutic dose was used in the HD group.

There was little or no effect between the SD and the HD groups when we assessed the severe disease and moderate disease cohort separately (OR 0.84 [95% CI 0.53–1.33] and OR 1.00 [95% CI 0.41–2.33], respectively) (Figure 2A).

We found no effect of HD compared with SD in the case of intermediate dose anticoagulation used as HD with OR 1.08 [95% CI 0.44–2.65]. In the case of therapeutic anticoagulation administered in the HD group, the pooled effect was 0.72 OR [95% CI 0.40–1.31], showing little or no effect. The overall pooled effect of the two cohorts was an OR of 0.85 [95% CI 0.55–1.31, *p* = 0.4237] (Figure 2B).

#### 3.3.2. Different D-Dimer Levels in the Included Trials

**Figure 3:**
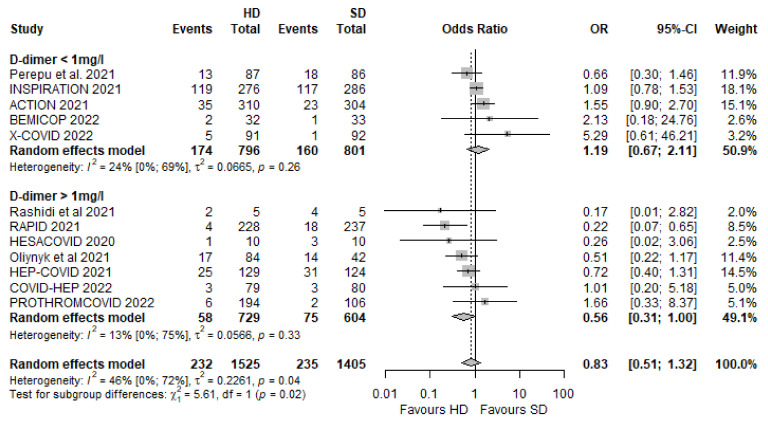
All-cause mortality in cohorts with different baseline D-dimer levels [19,21,22,23,24,25,50,52,53,54,55,56].

The cohort with D-dimer levels <1 mg/mL [22,23,24,52,54] showed no significant difference between the HD and the SD groups (OR 1.19 [95% CI 0.67–2.11]). Although nonsignificant, there is a tendency towards a decreased number of events in the HD group (OR 0.56 [95% CI 0.31–1.00]) in the case of the cohort with D-dimer >1 mg/mL [19,21,25,50,53,54,56]. The overall effect in the cohorts was an OR of 0.83 [95% CI 0.51–1.32, *p* = 0.3914] (Figure 3).

### 3.4. Any Thrombotic Events

**Figure 4:**
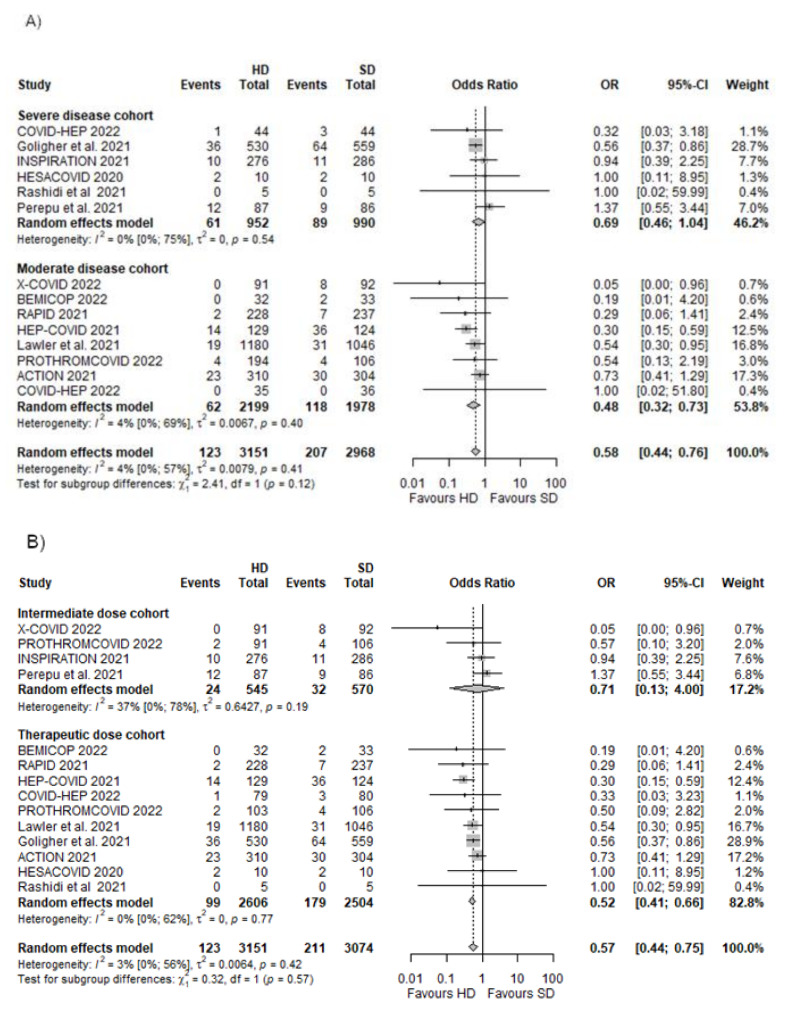
Any thrombotic events in different dosing regimens in moderate and severe disease cohorts [19,20,21,22,23,24,25,51,52,53,54,55,56]. (**A**) Any thrombotic events in severe and moderate disease cohorts; (**B**) any thrombotic events in cohorts where an intermediate or therapeutic dose was used in the HD group.

We included 13 RCTs [19,20,21,22,23,24,25,50,51,52,53,55,56] to assess the net effect regarding any thrombotic events, which consisted of any venous or arterial thrombotic events. This outcome covered a total of 6119 patients, out of which 123 patients in the HD group and 207 patients in the SD group suffered an adverse outcome. The overall effect size was an OR of 0.58 [95% CI 0.44–0.76, *p* = 0.0000], which indicates a significant difference favouring HD (Figure 4A).

#### Different Dosing Regimens in Moderate and Severe Disease

In the severe disease cohort, there was a numerical nonsignificant association of reducing the odds of thrombotic events in the HD group (OR 0.69 [95% CI 0.46–1.04]). This tendency favouring HD is associated with a significant decrease in thrombotic complications in the case of the moderate disease cohort with an OR of 0.48 [95% CI 0.32–0.76] (Figure 4A).

In the intermediate dose cohort, there was an association of a decreased number of thrombotic events in the HD group, but this did not reach statistical significance (OR 0.71 [95% CI 0.13–4.00]). Nevertheless, there was a significant association of reduction of thrombotic complications when patients were anticoagulated with therapeutic doses (OR 0.52 [95% CI 0.41–0.66]) (Figure 4B).

### 3.5. Major Bleedings

**Figure 5:**
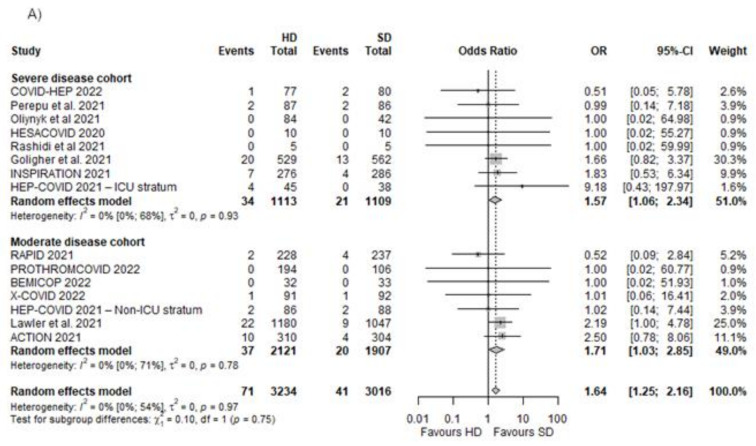

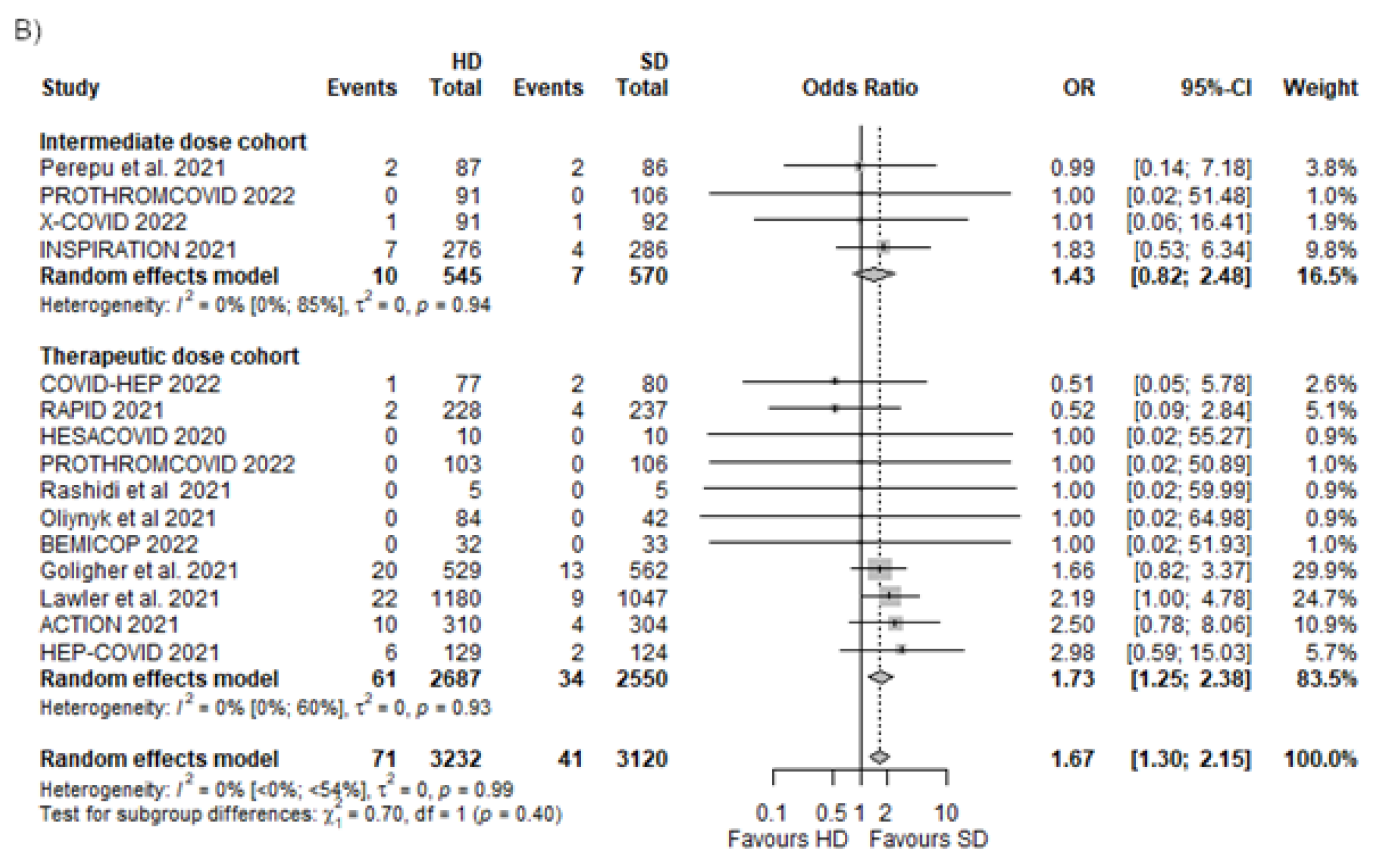
Major bleedings in different dosing regimens in moderate and severe disease cohorts [20,21,22,23,24,25,50,51,52,53,54,55,56]. (**A**) Major bleeding events in severe and moderate disease cohorts; (**B**) major bleeding events in cohorts where intermediate or therapeutic dose was used in the HD group.

We included 14 RCTs covering 6250 patients [19,20,21,22,23,24,25,50,51,52,53,54,55,56]. We found a significant association of increased risk of bleedings in the case of the HD group compared with the SD group (OR 1.64 [95% CI 1.25–2.16], *p* < 0.0017) (Figure 5).

#### Different Dosing Regimens in Moderate and Severe Disease

We found similar results favouring SD in severe and moderate disease cohorts (OR 1.57 [95% CI 1.06–2.34] and OR 1.71 [95% CI 1.03–2.85], respectively), both associated with an increased likelihood of the occurrence of bleedings (Figure 5A).

The association with increased major bleeding events did not reach the level of significance in the cohort where intermediate doses were administered in the HD group (OR 1.43 [95% CI 0.82–2.48]), although there was a significant association of increased likelihood of bleeding in the cohort with therapeutic anticoagulation used as HD (OR 1.73 [95% CI 1.25–2.15] (Figure 5B).

### 3.6. Progression of Disease

In order to examine the effect of different anticoagulant doses on disease progression, we used the following outcomes: the need for invasive mechanical ventilation, the need for ICU admission, progression to ARDS and an exploratory composite outcome of death, pulmonary embolism, and the need for invasive mechanical ventilation.

#### 3.6.1. Need for Invasive Mechanical Ventilation

**Figure 6:**
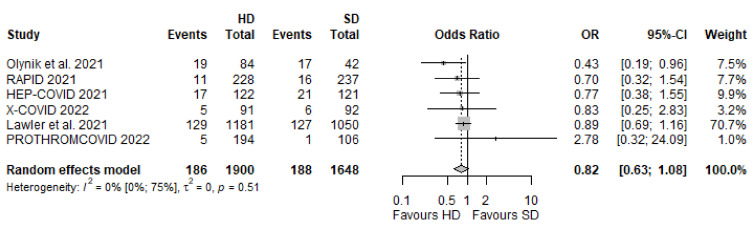
Need for invasive mechanical ventilation [19,20,21,23,25,54].

Data from six RCTs [19,20,21,23,25,54] were included in this analysis covering 3548 patients. There was no significant difference between the HD and SD groups regarding the number of patients who needed intubation, although a tendency towards a lower likelihood of occurrence of this event could be seen (OR 0.82 [95% CI 0.63–1.08], *p* = 0.1214) (Figure 6).

#### 3.6.2. Need for ICU Admission

Data from four RCTs were used in this analysis [21,24,25,53]. The need for ICU admission showed no significant difference between the HD and SD groups (OR 0.98 [95% CI 0.65–1.45, *p* = 0.9047] (Figure S2 in Supplementary Material).

#### 3.6.3. Progression to ARDS

Three RCTs reported this outcome [19,23,25]. Progression to ARDS occurred in 16 cases out of 412 in the higher dose cohort and 10 out of 319 in the standard dose cohort. The overall effect was 1.22 OR [95% CI 0.35–4.24, *p* = 0.7563], which did not reach the significance level (Figure S3 in Supplementary Material).

#### 3.6.4. Death, Pulmonary Embolism, and Need for Invasive Mechanical Ventilation

In the exploratory composite outcome, we pooled data from four RCTs [19,21,23,54]. When the numbers of death, pulmonary embolism, and intubations are taken together, there is a significant difference between the HD and SD groups, because, in the HD group, the event was less likely to occur (OR 0.46 [95% CI 0.21–0.67], *p* < 0.0001) (Figure S4 in Supplementary Material).

### 3.7. Assessment of Quality of Evidence

The risk of bias was assessed using the RoB2 tool [32,33]. The overall risk of bias was low in three of the RCTs [21,23,53]. One trial had a high risk because of missing outcome data [24]. There were some concerns in the case of the overall risk of other trials (Figure S5 in Supplementary Material).

**Figure 7:**
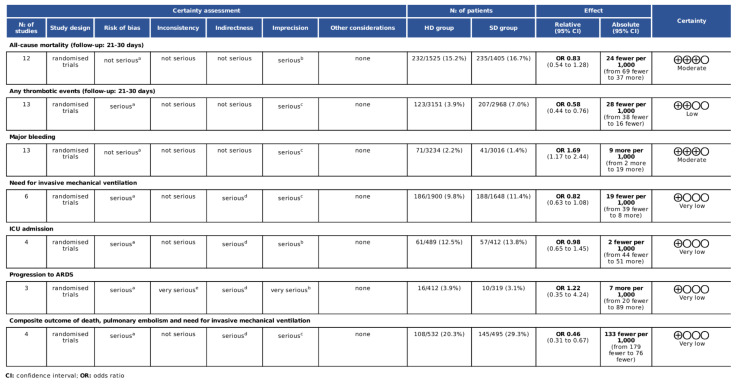
GRADE assessment of the included studies. ^a^ The included studies are open-label studies, some of them with blinded adjudication. All-cause mortality and major bleeding are objective outcomes are thus less influenced by a lack of allocation concealment. ^b^ Some studies included a small sample size and low number of events. The 95% confidence interval is wide and crosses the line of no effect. ^c^ Some of the studies included a small sample size and low number of events. ^d^ There were some differences in outcome measures. Need for invasive mechanical ventilation, ICU admission, progression to ARDS, and the composite outcome included a subjective clinical decision—this and the open-label design could have influenced the measurement of the outcomes. ^e^ There were wide differences in point estimates across studies.

The GRADE assessment resulted in “moderate” certainty owing to imprecision for all-cause mortality and major bleedings, while any thrombotic events were downgraded as “low” owing to the risk of bias and imprecision. The need for invasive mechanical ventilation, the need for ICU admission, and the composite outcome were qualified as “very low” certainty owing to serious risk of bias, indirectness, and imprecision. Progression to ARDS also resulted in “very low” certainty owing to the serious risk of bias, indirectness, very serious inconsistency, and imprecision (Figure 7).

## 4. Discussion

The COVID-19 pandemic has shocked the world and highlighted the importance of up-to-date knowledge. New treatments and old repurposed ones were demanded to curb the numbers of recently infected and dead.

In this meta-analysis, we analysed 14 RCTs [19,20,21,22,23,24,25,50,51,52,53,54,55,56], including a total number of 6253 patients, comparing the effects of HD with those of SD anticoagulation on mortality, any thrombotic events, major bleedings, and the progression of the disease in COVID-19 patients.

### 4.1. Different Dosing Regimens and Disease Severity

The ACTIV4-B trial [57] reported a low number of cardiopulmonary hospitalizations, thus it was halted. However, according to their findings, the use of apixaban 2.5 mg twice daily compared with apixaban 5 mg twice daily showed no benefit in the case of outpatients with a mild disease, as the authors found no effect on mortality or cardiopulmonary hospitalizations. Therefore, they concluded that the use of HD compared with SD seems futile in the case of a mild disease.

According to our results, the HD group compared with the SD group was not associated with lower mortality in all hospitalised COVID-19 patients (OR 0.83 [95% CI 0.54–1.28]). Although it was associated with a decreased risk of thrombotic events (OR 0.58 [95% CI 0.44–0.76]), it was parallelly associated with an increased chance of major bleeding (OR 1.64 [95% CI 1.25–2.16]). Therefore, we cannot exclude that the positive effects on thrombotic events were counteracted by the increased risk of major bleedings, thus resulting in no significant effect on mortality. Moreover, mortality in COVID-19 might have other causes that cannot be influenced by anticoagulation. On the other hand, owing to the short follow-up period of most of the included studies, we cannot assess the long-term benefits of anticoagulation in the case of these patients [59].

When we analysed the moderate and severe disease cohorts separately, we did not find significant differences in mortality (OR 1.00 [95% CI 0.41–2.44] and OR 0.84 [95% CI 0.53–1.33], respectively) in the HD group compared with the SD group.

In the moderate disease cohort, the effect of therapeutic dose anticoagulation compared with SD on thrombotic events was associated with a significant decrease in the incidence of any thrombotic events (OR 0.52 [95% CI 0.41–0.66]) and a nonsignificant tendency of decreasing all-cause mortality (OR 0.72 [95% CI 0.40–1.31]) compared with SD. Regarding the intermediate dose cohort as the HD group, we did not find a statistically significant effect regarding mortality (OR 1.08 [95% CI 0.44–2.65]) or thrombotic events (OR 0.71 [95% CI 0.13–4.00]).

The therapeutic dose anticoagulation was associated with a significant decrease in thrombotic events in the moderate disease cohort, but in a parallel way, it was associated with an increased risk of major bleedings (OR 1.73 [95% CI 01.25–2.38]). HD anticoagulation also significantly increased the risk of major bleeding in the severe disease cohort (OR 1.57 [95% CI 1.06–2.34]), but without a statistically significant benefit regarding the prevention of thrombotic events (OR 0.69 [95% CI 0.46–1.04]).

We have to take into consideration that some of the larger studies in severe disease cohorts used intermediate doses in the HD group [51,54], but only a minority of patients were administered intermediate doses in the moderate disease cohort [23,25].

It is important to note that a higher incidence of PE than deep vein thrombosis (DVT) was found in critically ill COVID-19 patients compared with non-COVID-19 patients, as reported by several studies [20,21,22,23,51,60]. These authors hypothesized that, in many instances, pulmonary artery occlusions in these patients could be interpreted as a manifestation of local pulmonary thrombi due to hyperinflammation rather than a consequence of an embolic event per se [23]. Pulmonary hyperinflammation in severe COVID-19 patients may be associated with hemostatic disorders such as hypercoagulation with fibrinolysis resistance [61,62,63,64,65], which can lead to organ failure due to thrombi in small vessels that theoretically cannot be prevented or treated with HD anticoagulation, although it could still be associated with an increased risk of bleeding events.

This assumption has been supported by recent multiplatform trials [20,51], which reported a significant increase in organ support-free days only in non-critically ill COVID-19 patients receiving a therapeutic dose of anticoagulation (OR 1.27 [95% CI 1.03–1.57]). Similar results were reported in the RAPID trial [21]. Unfortunately, outcomes of organ support-free days and ventilator-free days were only reported in three of the eligible RCTs [20,21,51], with different adjustment of statistical calculations; hence, the data were ineligible to be pooled together in the current meta-analysis (Supplementary Material Table S2). Therefore, we decided to analyse data based on disease progression such as the need for invasive mechanical ventilation, progression to ARDS, and need for ICU admission According to our result, therapeutic dose anticoagulation in a cohort with mostly moderately ill patients showed a slight tendency towards a reduction in the intubation rate (OR 0.82 [95% CI 0.63–1.08]). Although the result is statistically nonsignificant, it suggests that HD anticoagulation might be suitable as a preventive measure regarding disease progression in selected moderately ill patients, rather than a treatment option in the severe disease cohort with already settled respiratory failure.

Nevertheless, the severity of the coagulation disorder in COVID-19 patients could also differ and might influence the effectiveness of anticoagulation therapy.

### 4.2. Differences in the Degree of COVID-19-Associated Coagulopathy

The mechanism responsible for COVID-19-associated coagulopathy has not been fully elucidated, but it seems that hypercoagulation with impaired fibrinolysis induced by endothelial dysfunction due to hyperinflammation and hypoxia has a pivotal role in critically ill patients [66,67,68].

Hypercoagulation may be indicated by increased D-dimer levels, which are associated with an increased mortality and risk of complications in COVID-19 [8,29,30,31]. For instance, Tang et al. [7] found significantly higher D-dimer levels in non-survivors compared with those who survived (2.12 μg/mL [IQR 0.77–5.27] and 0.61 μg/mL [IQR 0.35–1.29], respectively, *p* < 0.001). Other studies had similar findings with significantly higher levels of D-dimer in patients with complications with PE [67,68,69].

In the multiplatform trials [20,51], D-dimer levels were used for risk stratification, but this did not result in significant variation in treatment effects between HD and SD anticoagulation. In these trials, patients were enrolled independently of their D-dimer levels and the median D-dimer levels were lower compared with those in some other studies [19,21,25,50,53,54,56], In the HEP-COVID trial on patients with more considerably elevated D-dimer levels, therapeutic-dose LMWH reduced not only the risk of thromboembolism, but also mortality, compared with low or intermediate-dose LMWH (absolute risk reduction, 13.2%) without increasing the risk of major bleeding (absolute incremental risk, 3.0%) [19]. In the pilot HESACOVID study, the use of therapeutic enoxaparin improved gas exchange over time and resulted in a higher ratio of successful liberation from mechanical ventilation in the case of severe COVID-19 patient with very high D-dimer levels (median: 4176 [1986–6365] μg/L); furthermore, D-dimer levels showed a significant decrease in the HD group (4176 [95% CI 1986–6365] μg/L vs. 1469 μg/L [95% CI 1034–1904] μg/L, *p* = 0.009), and a significant increase in the SD group (3408 μg/L [95% CI 1283–5532] μg/L vs. [95% CI 2291–7465]), *p* = 0.004) [50].

Therefore, D-dimer thresholds have been proposed to identify high-risk patients and guide decisions regarding anticoagulation on whether a therapeutic or prophylactic dose should be applied [69,70,71,72]. In a recently published meta-analysis, the weighted mean difference of D-dimer was 0.97 μg/mL (95% CI 0.65–1.29) between mild and severe groups of patients hospitalized with COVID-19; therefore, we performed a subgroup analysis of included RCTs with D-dimer levels below and above 1 µg/mL [31]. We found that, although none of the cohorts were associated with statistically significant effects, there was a clinically relevant tendency of decreased likelihood of death in the HD group in the cohort admitted to hospital with a D-dimer level > 1 µg/mL. In order to investigate whether HD anticoagulation prevented thrombotic events and the progression of disease with elevated D-dimer levels in a cohort consisting mostly of moderately ill patients, we performed an exploratory composite outcome that included death, the incidence of pulmonary emboli, and the need for mechanical ventilation. We found statistically significant results favouring higher dose thromboprophylaxis (OR 0.46 [95% CI 0.31–0.67], *p* < 0.0001). On the basis of our results, one may assume that therapeutic dose anticoagulation could be beneficial in moderately ill patients with elevated D-dimer levels.

### 4.3. Strengths and Limitations

To date, this is the most up-to-date and comprehensive meta-analysis on the effect of different anticoagulant regimens on COVID-19 infection. We included traditional outcomes that assess the effect of different anticoagulation doses on thrombotic events and bleeding, but included additional clinical outcomes that point towards the course of the disease by comparing higher and standard dose thromboprophylaxis. Furthermore, we analysed the interplay of baseline D-dimer levels on the effect of anticoagulation on all-cause mortality. We defined a selected subpopulation of patients, the moderately ill with elevated D-dimer levels, in which the progression of the disease may be prevented by therapeutic anticoagulation. This had not been reported before. We also tried to minimize the effects of confounding factors present in different trials by conducting leave-one-out analyses on all outcomes that had more than five trials included (Supplementary Material Figures S6–S13).

Our study has several limitations. First, data extraction resulted in some compromise as Lawler et al. published survival without intubation through 28 days as the number of patients per total number, but we chose to calculate the number of patients who were intubated or died and pooled together with the number of patients who needed intubation, as this was an outcome to evaluate the progression of disease [20]. In addition, in the case of composite outcomes, we cannot exclude that there are overlaps in the patient population. Second, the low number of studies and the clinical heterogeneity of the population make it difficult to address the question of thromboprophylaxis, as the difference in anticoagulant regimens, dose, duration, and the trial population may work as confounding factors. Third, there are differences in ICU admission among countries. Fourth, some trials enrolled a low number of participants, thus a low event rate was reported, which may have influenced our analysis [50,56]. Besides, some preplanned analyses could not be performed because of the differences in statistical adjustments between the trials. Finally, we included an article that was not peer-reviewed [25].

## 5. Conclusions and Implications for Practice and Research

Our analysis including recently published RCTs [25,53,54,56] confirmed the main finding of previous meta-analyses [73,74,75,76,77,78,79] and endorsed the current guidelines (Table S3 in Supplementary Material) [80,81,82,83]. On the basis of our results, we cannot advocate for the routine use of therapeutic dose thromboprophylaxis in all COVID-19 patients either. Nevertheless, there is a tendency that favours HD regarding disease progression, specifically therapeutic dose anticoagulation with UFH or LMWH, in patients with considerably elevated D-dimer levels who did not need ICU level care at hospital admission.

Further results of ongoing studies are needed to define more precisely at what time it is appropriate to start higher dose thromboprophylaxis and in which selected patients it should be applied. However, there is a great risk of the populations of these trials being notably heterogeneous, as diverse variants of the SARS-CoV2 virus have emerged, new therapeutic options were implemented, and rates of immunizations varied between different countries and periods. Thus, what seemed beneficial in the earlier wave of the pandemic with considerable thrombotic complications reported might have less importance later, as these might serve as confounding factors that influenced the presentation of the disease.

## Figures and Tables

**Table 1:** Characteristics of included studies.

Study	Design	Enrollment Period	Country	Sample Size	Patients Admitted to	Intervention Arms	Follow-Up Period (days)
HESACOVID 2020 [50]	Open-label, single center RCT	From April 2020 to July 2020	Brazil	20	100% ICU	Therapeutic: enoxaparin 1 mg/kg twice daily or UFH adjusted to aPTT 1.5–2 Standard: enoxaparin 40 mg once daily or UFH 5000 three times a day ^a^	28
Goligher et al., 2021 [51]	Open-label, adaptive, multiplatform RCT	From 21 April 2020 to 19 December 2020	United States, Canada, the United Kingdom, Brazil, Mexico, Nepal	1103	100% ICU	Therapeutic: enoxaparin 1.5 mg/kg once daily, UFH titrated to have 1.5–2.5 aPTT, dalteparin 200 UI once daily Tinzaparin 175 UI/kg once daily Standard: enoxaparin 40 mg once daily, dalteparine 5000 UI once, tinzaparine 4500 UI, fondaparinux 2.5 mg, UFH 5000 UI/ 8–12 h ^b^	21
Lawler et al., 2021 [20]	Open-label, adaptive, multiplatform RCT	From 21 April 2020 to 22 January 2021	United States, Canada, the United Kingdom, Brazil, Mexico, Nepal	2219	100% ward	Therapeutic: enoxaparin 1.5 mg/kg once daily, UFH titrated to have 1.5–2.5 aPTT, dalteparin 200 UI once daily OR 100 UI twice daily tinzaparin 175 UI/kg once daily Standard: enoxaparin 40 mg once daily, dalteparine 5000 UI once, tinzaparine 4500 UI, fondaparinux 2.5 mg UFH 5000 UI/ 8–12 h ^b^	21
Perepu et al., 2021 [52]	Open-label, multicenter RCT	From 26 April 2020 to 6 January 2021	USA	173	38% ward 62% ICU	Intermediate: enoxaparin 1 mg/kg SC daily Standard: enoxaparin 40 mg daily ^b^	30
X-COVID 2021 [23]	Open-label, multicentre RCT	From 30 April 2020 to 25 April 2021	Italy	183	100% ward	Intermediate: enoxaparin 40 mg twice daily Standard: enoxaparin 40 mg once daily	30
COVID-HEP 2022 [53]	Open-label, multicentre RCT	From April 2020 to June 2021	Switzerland	159	45% ward 26% intermediate care 28% ICU	Therapeutic: enoxaparin 1 mg/kg twice daily or UFH with anti-Xa titration Standard: enoxaparin 40 mg once daily or UFH 5000 IU twice daily at ward enoxaparin 40 mg twice daily or UFH 5000 three times daily ^c^	30
HEP-COVID 2020 [19]	Single-blinded, multicentre RCT	From 8 May 2020 to 14 May 2021	USA	253	67% ward 32% ICU	Therapeutic: enoxaparin 1 mg/kg twice daily Standard dose: enoxaparin 30 or 40 mg once or twice daily ^d^	30
RAPID 2021 [21]	Open-label, adaptive, multicentre RCT	from 29 May 2020 to 12 April 2021	Brazil, Canada, Ireland, Saudi Arabia, United Arab Emirates, United States of America	465	100% ward	Therapeutic: enoxaparin 1.5 mg/kg once daily, dalteparin 200 UI/kg once daily, tinzaparin 175 IU/kg once daily, or UFH titrated according to aPPT Standard: 40 mg enoxaparin once daily, dalteparin 5000 UI daily, tinzaparin 4500 UI daily, UFH 5000 UI two or three times a day ^b^	28
ACTION 2021 [22]	Open-label, pragmatic, multicentre RCT	From 24 June 2020 to 26 February 2021	Brazil	615	93% ward 6% ICU	Therapeutic: Rivaroxaban 20 mg once daily, enoxaparin 1 mg/kg twice daily, UFH titrated until anti-Xa 0.3–0.7 Standard: local prophylatic guidelines	30
Oliynyk et al., 2021 [54]	Open-label RCT	From July 2020 to 1 March 2021	Ucraine	126	100% ICU	Therapeutic: group 1: enoxaparin of 100 antiXa IU/kg twice daily group 2: UFH titrated to aPTT 40–70 s Standard: enoxaparin of 50 antiXa IU/kg once daily	28
INSPIRATION 2021 [55]	Open-label, multicenter RCT	From 29 July 2020 to 19 November 2020	Iran	562	100% ICU	Intermediate: enoxaparin 1 mg/kg daily Standard: enoxaparin 40 mg daily	30
Rashidi et al., 2021 [56]	Open-label, pilot RCT	From September 2020 to April 2021	Iran	10	100% ICU	Therapeutic dose: UFH 5000 IU every 8 h Standard: UFH titrated until aPTT 5–70	30
BEMICOP 2022 [24]	Open-label, multicenter, RCT	From October 2020 to May 2021	Spain	65	100% ward	Therapeutic: bemiparin 115 UI/kg daily Standard: bemiparin 3500 UI daily	30
PROTHROMCOVID 2022 [25]	Open-label, multicenter RCT	From 1 February 2021 to 30 September 2021	Spain	300	100% ward	Therapeutic: tinzaparin 175 UI/kg Intermediate: tinzaparin 100 UI/kg Standard: tinzaparin 4500 UI daily	30

Abbreviations used in this table:RCT: randomised controlled trial, BMI: body mass index, CrCl: creatinine clearance, UFH: unfractioned heparine, UI: international units. Explanations: ^a^ adjusted to age, BMI, and CrCL; ^b^ adjusted to BMI; ^c^ adjusted to patient’s weight; ^d^ adjusted for CrCl.

## Data Availability

Data sharing not applicable.

## Supplementary Materials

The following supporting information can be downloaded at https://www.mdpi.com/article/10.3390/biomedicines10092194/s1, **Figure S1:** PRISMA 2020 flow diagram of the initial search, **Table S1:** Detailed inclusion and exclusion criteria of included trials with baseline D-dimer levels, **Figure S2:** Need for ICU admission, **Figure S3:** Progression to ARDS, **Figure S4:** Composite outcome of death, pulmonary embolism and need for invasive mechanical ventilation, **Figure S5:** Risk of bias assesment 2 in the included trials, **Table S2:** Other clinical outcomes, **Table S3:** Latest recommendations of guidelines on anticoagulation in patients infected with COVID-19, **Figure S6:** All-cause mortality in cohorts with different disease severity, **Figure S7:** All-cause mortality in cohorts with different anticoagulant doses, **Figure S8:** All-cause mortality in cohorts with different baseline d-dimer levels, **Figure S9:** Any thrombotic events in cohorts with different disease severity, **Figure S10:** Any thrombotic events in cohorts with different anticoagulant doses, **Figure S11:** Major bleeding events in cohorts with different disease severity, **Figure S12:** Major bleeding events in cohorts with different anticoagulant doses, **Figure S13:** The need for invasive mechanical ventilation.
