# Supplementary material for: Higher Dose Anticoagulation Cannot Prevent Disease Progression in COVID-19 Patients: A Systematic Review and Meta-Analysis
Source: Biomedicines. 2022 Sep 5;10(9):2194. doi: 10.3390/biomedicines10092194 (PMC9496532; doi:10.3390/biomedicines10092194)
Supplement: Supplementary file 1 [file biomedicines-10-02194-s001.zip › biomedicines-1892528-supplementary.pdf]

Figure S1: PRISMA 2020 flow diagram of the initial search

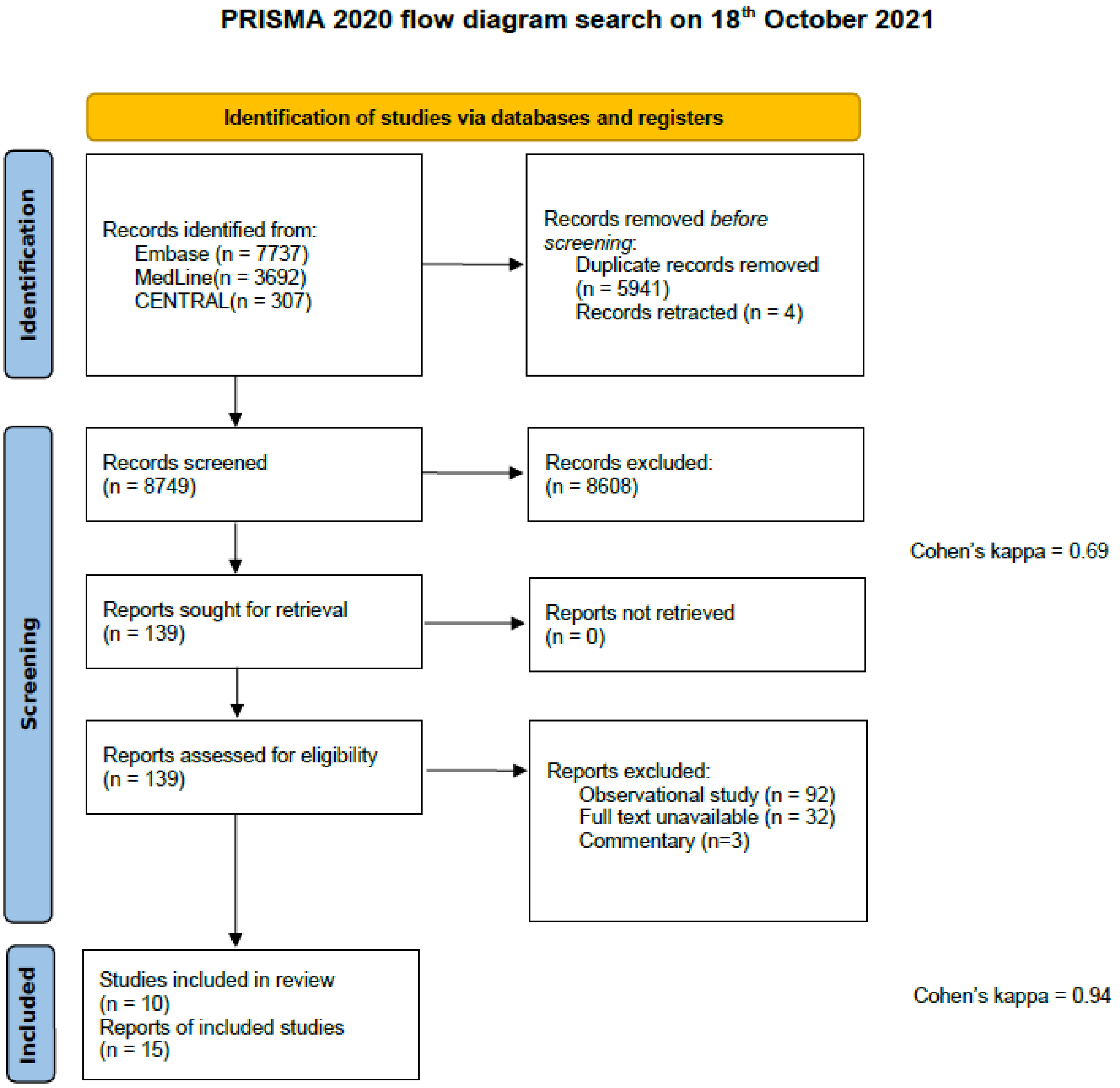

Table S1.: Detailed inclusion and exclusion criteria of included trials with baseline D-dimer levels

Study	Inclusion criteria	Exclusion criteria	Median baseline D-dimer in ng/ml (IQR) SD/HD group	Mean age (Standard Deviation) SD/HD group
HESACOVID 2020	Adults with laboratory confirmed SARS-CoV-2 infection, presence of ARDS, severe clinical presentation with respiratory failure requiring mechanical ventilation, D-dimer levels greater than 1000 µg/L; prothrombin time/international normalized ratio (INR) < 1.5; activated partial thromboplastin time (aPTT)/ratio < 1.5, and platelet count greater than 100,000/mm3	Creatinine clearance (CrCl) < 10 mL/min, severe circulatory shock with a dose of norepinephrine higher than 1.0 µg/kg/min, chronic renal failure in renal replacement therapy Child B and C chronic liver dis- ease, advanced diseases, such as active cancer, heart failure with functional NYHA class III and IV , COPD using home oxygen, advanced dementia, significant disability from stroke	3408 (1283-5532) / 4176 (1986-6365)*	55(16) / 58(10)

		or severe head injury, car- diorespiratory arrest, pregnant women, recent major surgery or severe trauma in the last 3 weeks, recent stroke in the last 3 months<br>Active bleeding, blood dyscrasia such as hemophilia, Von Willebrand factor deficiency<br>Clinical indication for therapeutic anticoagulation due to pulmonary embolism, and acute coronary syndrome		
Goligher et al. 2021	Adults hospitalised for COVID-19 wih severe disease (requiring ICU)	Admission to the ICU with Covid-19 to a hospital for 72 hours or less before randomization<br>At imminent risk for death and there was no ongoing commitment to full organ support<br>High risk for bleeding, dual antiplatelet therapy, clinical indication for therapeutic dose anticoagulation, or history of heparin sensitivity.	890 (430-1740) / 823 (386.2-1844.2)	61.7 (12.5) / 60.4 (13.1)
Lawler et al. 2021	Adults hospitalised for Covid-19 infection with moderate disease (without the need for ICU-level care)	Admission to the ICU with Covid-19 to a hospital for 72 hours or less before randomization<br>At imminent risk for death and there was no ongoing commitment to full organ support<br>High risk for bleeding, dual antiplatelet therapy, clinical indication for therapeutic dose anticoagulation, or history of heparin sensitivity.	750 (500-1350) / 800 (450-1250)	58.8 (13.9) / 59 (14.1)
Perepu et al. 2021	Adultss hospitalised with diagnosis of COVID-19 admitted to an ICU and/or had a modified ISTH Overt DIC score ≥3	Clinical indication for therapeutic dose anticoagulation<br>Active major bleeding, severe thrombocytopenia (platelet count <25,000/µL), within the prior 3 months,<br>Acute or chronic renal insufficiency with an estimated creatinine clearance <30 ml/min.	950 (435-1990) / 785 (515-2025)	63.5 (30-85) / 65 (24-86)**
X-COVID 2021	All patients aged >18 years with COVID-19 infection.	Directly admitted to ICU<br>Creatinine clearance <15 ml/min/1.73 m2,<br>Clinical indication for therapeutic anticoagulant treatment for prior indications, were on treatment with heparin at higher doses than recommended for thromboprophylaxis,<br>Active bleeding or at high bleeding risk	320 (180-710) / 360 (190-680)	59 (48-72) / 60(53-73)**
COVID-HEP 2022	Acute severe laboratory confirmed COVID-19 infection. (D-dimer>1000 ng/ml in ward, or hospitalization in intermediate	Therapeutic anticoagulation for any indication other than COVID-19, contraindication to therapeutic anticoagulation, a high risk of bleeding, ongoing pregnancy, extreme body weight, and participation in another clinical trial.	1678 (1441) / 1706 (1540) ***	62.9 (12.2) /62.1 (11.9)
HEP-COVID 2020	Hospitalized nonpregnant adults 18 years or older with diagnosed with COVID-19, with requirement for	Clinical indication for full-dose anticoagulation or dual anti- platelet therapy	1700 (1072-2942) /	67.7 (14.1) / 65.8 (13.9)

	supplemental oxygen per investigator judgment and plasma D-dimer level greater than 4 times the upper limit of normal based on local laboratory criteria or a sepsis-induced coagulopathy score of 4 or greater	Bleeding within the past month, active gastrointestinal or intracranial cancer<br>Bronchiectasis or pulmonary cavitation, hepatic dysfunction with baseline inter-national normalized ratio greater than 1.5,<br>Creatinine clear- ance (CrCl) less than 15 mL/min/1.73 m2,<br>Platelet count less than 25 000/µL, a history of heparin-induced thrombocytopenia (HIT)	1451 (1045-3393)	
RAPID 2021	Adults with laboratory confirmed SARS-CoV-2 infection and elevated D-dimer levels within the first 5 days of admission. D-dimer levels were required to be above the upper limit of normal (ULN) of the local hospital in the presence of an oxygen saturation ≤93% on room air, or ≥2 times the ULN irrespective of oxygen saturation.	Substantial bleeding risks,<br>Clinical indication for or any contraindication against heparin anticoagulation<br>If they had already met, or would imminently meet any component of the primary outcome.	1250 (450)<br>/<br>1050 (350)***	59.6 (15.5)<br>/<br>60.4 (14.1)
ACTION 2021	Adults hospitalised with a confirmed diagnosis of COVID-19, with symptoms for up to 14 days before randomisation, and elevated D-dimer concentration (above the upper limit of normal reference range per local laboratory).	Clinical indication for therapeutic anticoagulation<br>Contraindications to rivaroxaban or heparin,<br>High risk for bleeding.	73% of patients had a value between 250-750 ng/ml, 27% more than 750 ng	56.5 (14.5)<br>/<br>56.7 (14.1)
Oliynyk et al. 2021	Confirmed COVID-19 with bilateral interstitial pneumonia on CT scan, respiratory failure (PaO2<60mmHg), D-dimer > 3mg/l, platelet count <120x10^9/L	Intubation prior to enrollment, contraindication to LMWH or UFH, active bleeding, CrCl<30 ml/min, unstable arterial hypertension, PE confirmed, pregnancy, breastfeeding,.	5246(3567-5657)<br>/<br>4494 (4221-5664)<br>/<br>5245 /(4221-5445)	71 (68-72) /<br>70.5 (68-72)**
INSPIRATION 2021	Adults admitted to the ICU with polymerase chain reaction testing–confirmed COVID-19 within 7 days of the index hospitalization	Life expectancy less than 24 hours<br>Clinical indication for therapeutic-dose anticoagulation<br>History of heparin-induced thrombocytopenia, platelet count less than 50 ×103/µL,<br>Overt bleeding	910 (410-2380)<br>/<br>1037 (460-3121)	61 (47-71)<br>/<br>62 (51-70.7)***
Rashidi et al. 2021	Adult patients with positive SARS-CoV-2<br>reverse transcription-polymerase chain reaction (RT-PCR) assay within<br>5 days of the index hospitalization without known diagnosis of throm bosis, who were admitted to the ICU with PaO2/FiO2 (P/F) ratios <100 and D-dimer levels >3000 ng/mL were assessed for eligibility	Hemodynamic instability, estimated life expectancy<24h, pregnancy or breastfeeding, Active bleeding and any contraindications for thrombolytic and anticoagulation therapy, allergy to study drug.	15,000 (7293–15,000)<br>/<br>10,000 (6340.5–10,000)	63 (48-65.5)<br>/<br>53 (43-67) ***
BEMICOP 2022	Adult patients who required admission due to non-severe (CURB65 ≤2 points and baseline oxygen saturation ≥90%) COVID-19 pneumonia, with baseline D-dimer >500 ng/mL	Clinical indication for full therapeutic-dose anticoagulation<br>Acute venous thromboembolism within prior 3 months	770 (590-1030)<br>/<br>780 (600-1125)	62.3 (12.2)<br>/<br>63 (13.7)

		Acute cardiovascular event within prior 3 months, acute stroke within prior 3 months<br>Active major bleeding or high risk of bleeding<br>Severe thrombocytopenia (<25,000/mm3) Acute or chronic renal insufficiency with estimated Creatinine Clearance< 30 ml/min calculated by the modified Cockcroft and Gault formula • Weight < 40 kg • Known allergies to ingredients contained in enoxaparin or allergy to heparin products such as history of heparin induced thrombocytopenia. • Current pregnancy		
PRO-THROMCOVID 2022	Adults with a body weight of 50-100 kg who required admission to a conventional (non-critical) hospital ward due to COVID-19 pneumonia were included if they also met any of the following criteria: a) baseline oxygen saturation ≤94%, b) D-dimer > 1000 µg/L, c) C Reactive Protein (CRP) > 150 mg/L, or d) interleukin-6 (IL6) > 40 pg/mL)	Need for full-dose anticoagulant therapy, active bleeding or situations prone to bleeding, glomerular filtration rate < 30 ml/min/1.73 m2, platelet count<80x109/L, previous heparin-induced thrombocytopenia, and hypersensitivity/intolerance to heparins.	6180 (3750-11000) / 6860 (4040-13400) / 6200 (3630-12000)	54.1 (15) / 56.5 (14) / 58.5 (14)

**Mean + 95% confidence intervals (95%CI), **Median + interquartile range (IQR), ***Mean and standard deviation (SD)*

Figure S2: Need for ICU admission

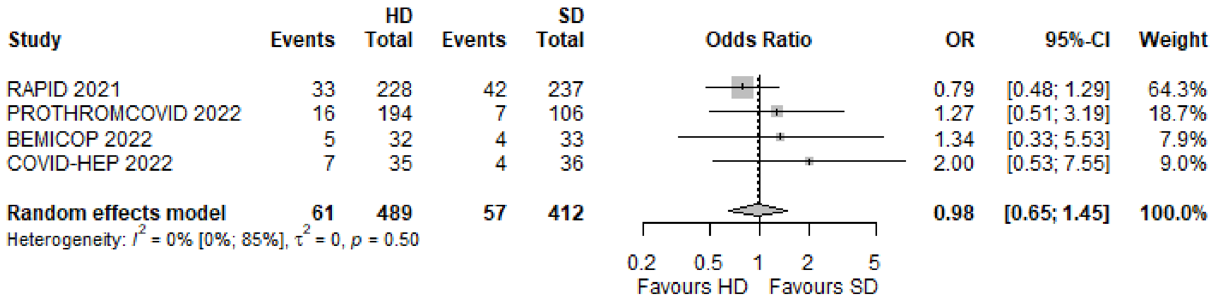

Figure S3: Progression to ARDS

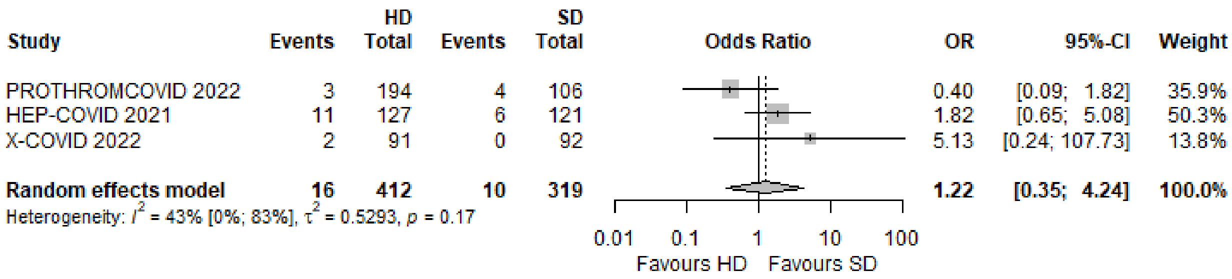

Figure S4: Composite outcome of death, pulmonary embolism and need for invasive mechanical ventilation

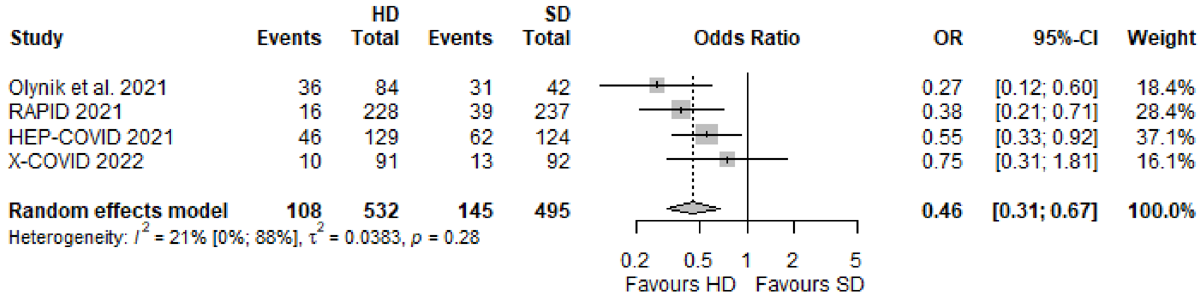

Figure S5: Risk of bias assesment 2 in the included trials

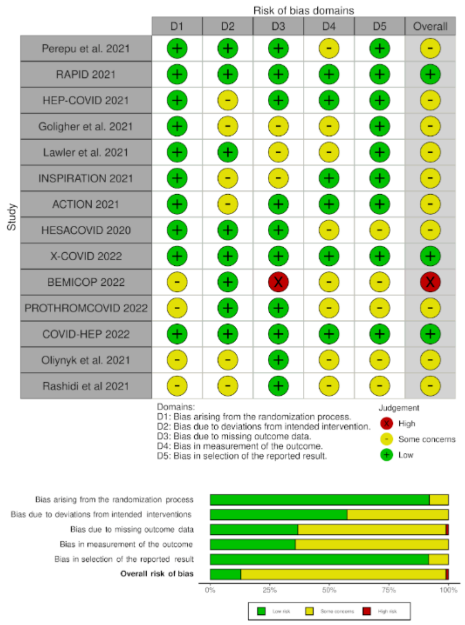

Table S2.: Other clinical outcomes

Study	HD group size	Median	IQR	SD group size	Median	IQR	OR	95%CI
**Organ support-free days**								
Goligher et al. 2021	536	1	(-1 – 16)	567	4	(-1 – 16)	0.83	0.67-1.03
Lawler et al. 2021	939			801			1.27	1.03-1.58
RAPID 2021	228	25.8*	6.2*	237	24.1*	8.8*	1.41	0.9-2.21
**Ventilator-free days**								
INSPIRATION 2021	276	30	(3-30)	286	30	(1-30)	1.06	0.76-1.48
HESACOVID 2020	10	15	(6-16)	10	0	(0-11)		
RAPID 2021	228	26.5*	5.6*	237	24.7*	8.5*		
Lawler et al. 2021	994			864			1.22	0.97-1.55
**Length of hospital stay**								
INSPIRATION 2021	276	5	(2-10)	286	6	(3-11)		
HESACOVID 2021	10	31	(22-35)	10	30	(23-38)		
Rashidi et al. 2021	5	8	(4-19)	5	11	(8-12)		
X-COVID 2022	91	11	(7-14)	92	8	(6-16)		
ACTION 2021	The number of wins was 28 899 (34·8%) in the therapeutic group and 34 288 (41·3%) in the prophylactic group (win ratio 0·86 [95% CI 0·59–1·22], p=0·40).							
Lawler et al. 2021							1.03	0.94-1.13
**Change in PaO2/FiO2 ratio from baseline to 7, 14 days;**								
**PaO2/FiO2 ratio at baseline**								
HESACOVID 2021	10	163	(133-193)	10	184	(146-222)		
**PaO2/FiO2 ratio after 7 days**								
HESACOVID 2021	10	209	(171-247)	10	168	(142-195)		
**PaO2/FiO2 ratio after 14 days**								
HESACOVID 2021	10	261	(230-293)	10	195	(128-262)		
**Change of PaO2/FiO2 ratio within 48 h of enrollment**								
Rashidi et al. 2021	5	-7.6	(-14.5-68.9)	5	-15.8	(-28-24.1)		

**The values are reported as mean and standard deviation*

Table S3: Latest recommendations of guidelines on anticoagulation in patients infected with COVID-19

	Guidelines
**American Society of Hematology**	"The ASH guideline panel suggests using therapeutic-intensity over prophylactic-intensity anticoagulation for patients with COVID-19-related acute illness who do not have suspected or confirmed VTE or another indication for anticoagulation. Patients with COVID-19-related acute illness are defined as those with clinical features that would typically result in admission to a medicine inpatient ward without requirement for intensive clinical support. Examples include patients with dyspnea or mild to moderate hypoxia (very low certainty). "[1]<br>The American Society of Hematology (ASH) guideline panel suggests using prophylactic-intensity over intermediate-intensity or therapeutic-intensity anticoagulation for patients with coronavirus disease 2019 (COVID-19)–related critical illness who do not have suspected or confirmed venous thromboembolism (VTE) Patients with COVID-19–related critical illness are defined as those suffering from an immediately life-threatening condition who would typically be admitted to an intensive care unit (ICU). Examples include patients requiring hemodynamic support, ventilatory support, and renal-replacement therapy. (very low certainty).[1(p19)]
International Society of Thrombosis and Hemostasis (ISTH)	"VTE prophylaxis in non-ICU hospitalized COVID-19 patients:<br>1.A universal strategy of routine thromboprophylaxis with standard-dose UFH or LMWH should be used after careful assessment of bleed risk, with LMWH as the preferred agent. Intermediate-dose LMWH may also be considered (30% of respondents).<br>2. VTE prophylaxis recommendations should be modified based on extremes of body weight, severe thrombocytopenia (ie platelet counts of 50 000 × 109 per liter or 25 000 × 109 per liter) or deteriorating renal function."[2]<br>"VTE prophylaxis in sick ICU hospitalized COVID-19 patients:<br>Routine thromboprophylaxis with prophylactic-dose UFH or LMWH should be used after careful assessment of bleed risk. Intermediate-dose LMWH (50% of respondents) can also be considered in high risk patients. Patients with obesity as defined by actual body weight or BMI should be considered for a 50% increase in the dose of thromboprophylaxis. Treatment-dose heparin should not be considered for primary prevention until the results of randomized controlled trials are available.<br>Multi-modal thromboprophylaxis with mechanical methods (ie, intermittent pneumonic compression devices) should be considered (60% of respondents)."[2]
National Institutes of Health (NIH)	"The Panel recommends against the use of antiplatelet therapy to prevent COVID-19 progression or death in noncritically ill patients<br>There is insufficient evidence for the Panel to recommend either for or against antiplatelet therapy in critically ill patients with COVID-19.<br>For adults who require low-flow oxygen and do not require intensive care unit (ICU)-level care: The Panel recommends using a therapeutic dose of heparin for patients with D-dimer levels above the upper limit of normal, who require low-flow oxygen, and who do not have an increased bleeding risk<br>For adults who require ICU-level care, including those receiving high-flow oxygen:<br>• The Panel recommends using a prophylactic dose of heparin as VTE prophylaxis, unless a contraindication exists.<br>• The Panel recommends against the use of an intermediate dose (e.g., enoxaparin 1 mg/kg once daily) or a therapeutic dose of anticoagulation for VTE prophylaxis, except in a clinical trial.<br>• For patients who start on a therapeutic dose of heparin in a non-ICU setting due to COVID-19 and then transfer to the ICU, the Panel recommends switching from the therapeutic dose to a prophylactic dose of heparin, unless VTE is confirmed"[3].
Anticoagulation Forum	"We recommend against any specifc antithrombotic preventative therapy for ambulatory (non-hospitalized) adult patients with mild COVID-19 infection who have no other indication for antithrombotic therapy.<br>We recommend that all patients hospitalized with COVID-19 receive at least standard dose thromboprophylaxis. We suggest that non-heparin anticoagulants (i.e., direct oral anticoagulants) be avoided when therapeutic intensity thromboprophylaxis is utilized.<br>We suggest that clinicians consider the use of therapeutic intensity LMWH or UFH thromboprophylaxis for non-critically ill patients at increased risk of disease progression or thromboembolism and who are not high risk for anticoagulantrelated bleeding<br>We recommend that adult patients who are critically ill at the time of hospitalization receive standard dose thromboprophylaxis instead of intermediate- or therapeutic intensity thromboprophylaxis."[4]
American College of Chest Physicians	"In acutely ill hospitalized patients with COVID-19, we recommend current standard dose anticoagulant thromboprophylaxis over intermediate (LMWH BID or increased weight-based dosing) or full treatment dosing, per existing guidelines.

In critically ill patients with COVID-19, we suggest current standard dose anticoagulant thromboprophylaxis over intermediate (LMWH BID or increased weight-based dosing) or full treatment dosing, per existing guidelines”[5]

Leave-one-out analyses

Figure S6: All-cause mortality in cohorts with different disease severity

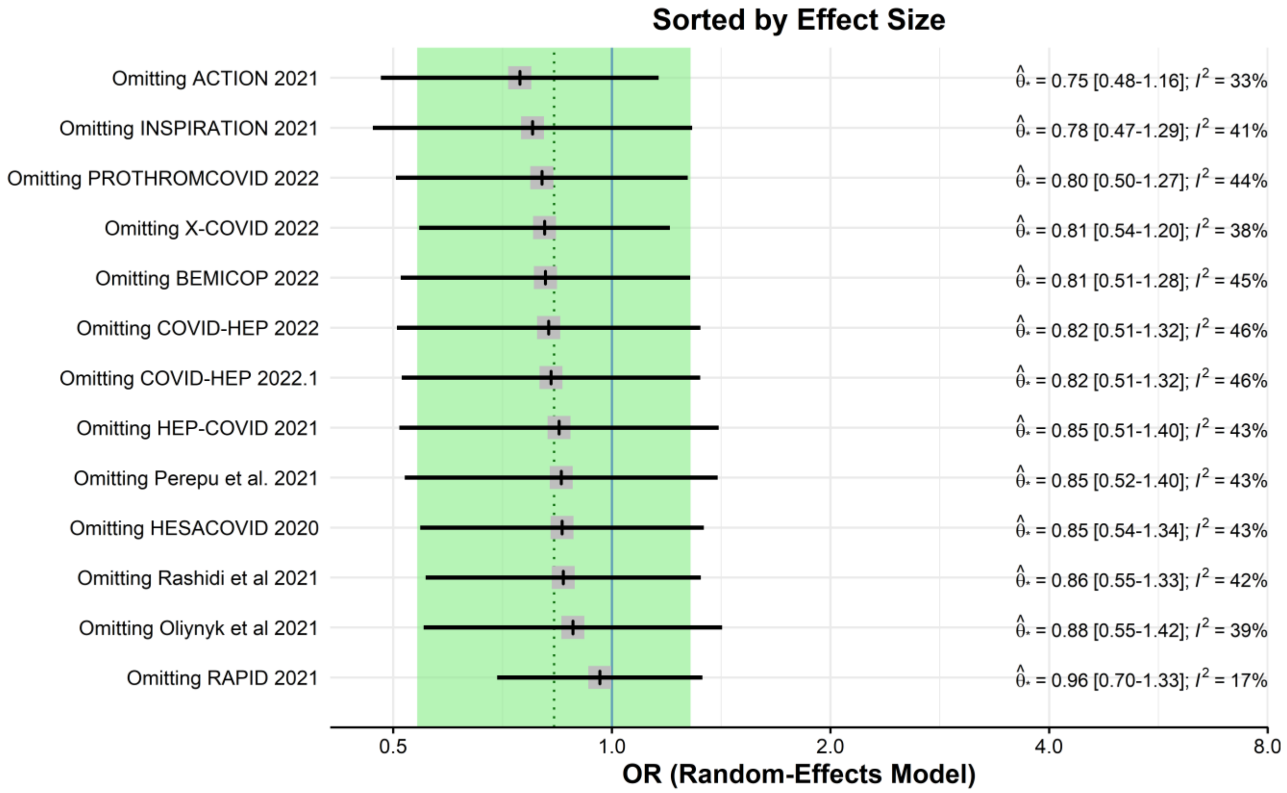

Figure S7: All-cause mortality in cohorts with different anticoagulant doses

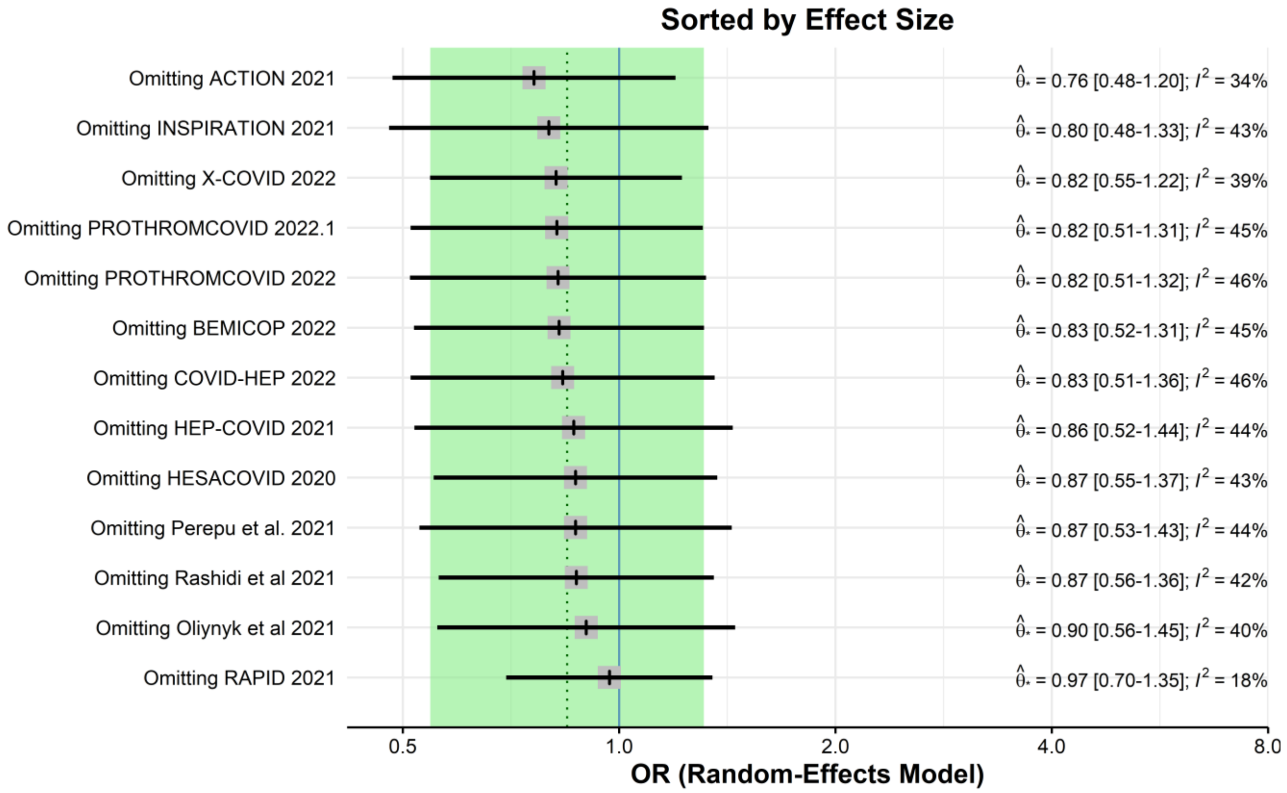

Figure S8: All-cause mortality in cohorts with different baseline d-dimer levels

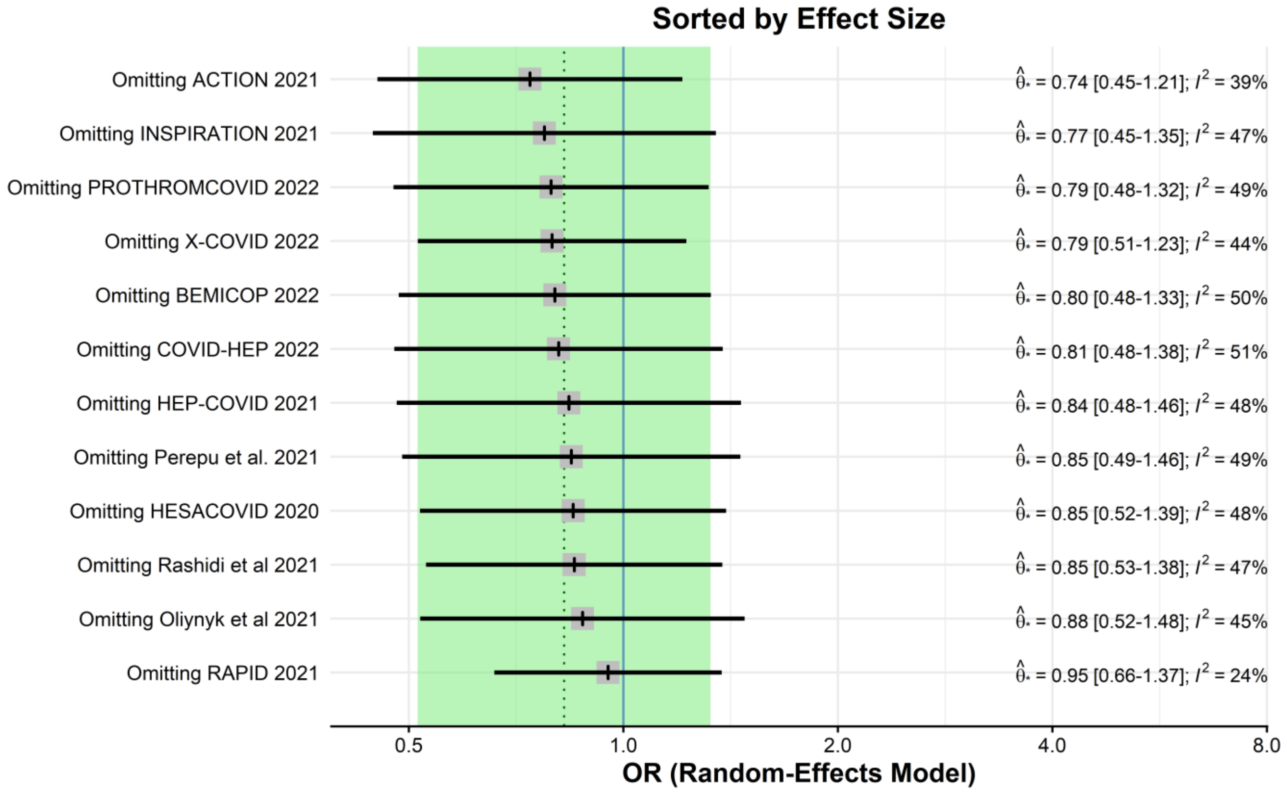

Figure S9: Any thrombotic events in cohorts with different disease severity

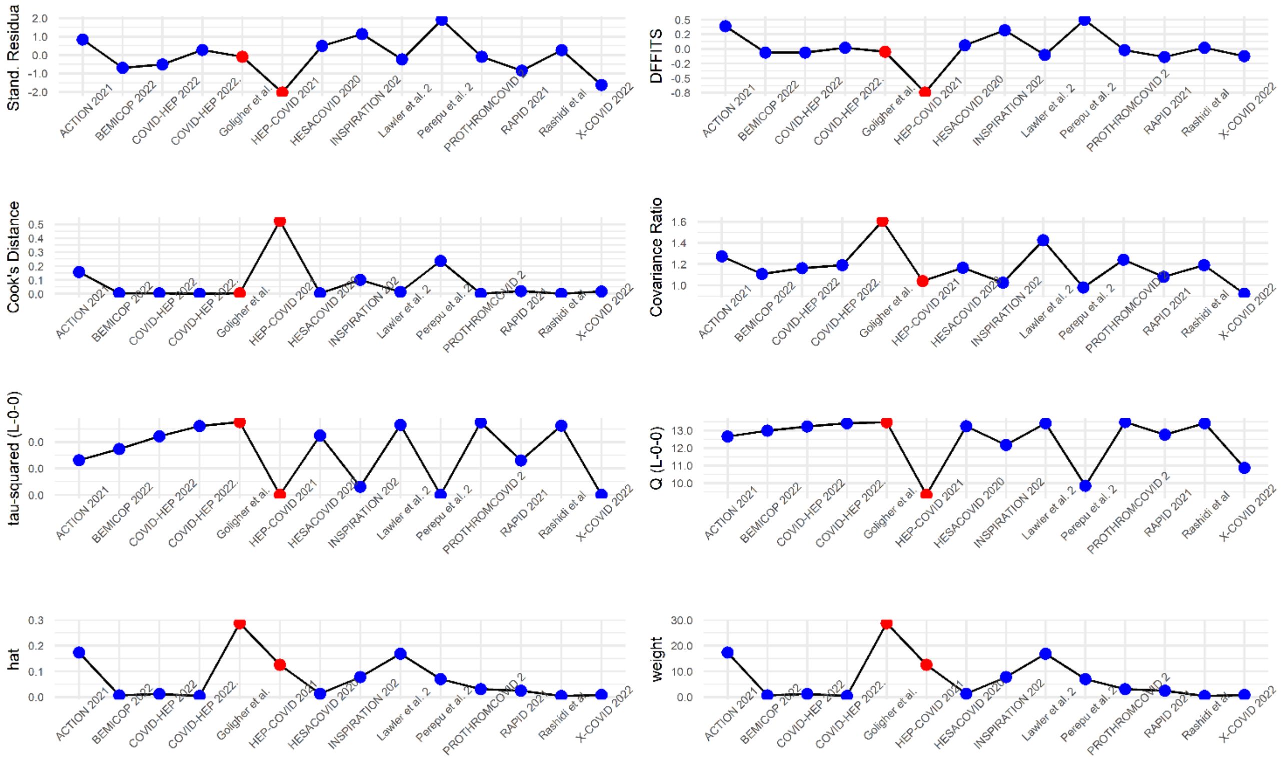

Figure S10: Any thrombotic events in cohorts with different anticoagulant doses

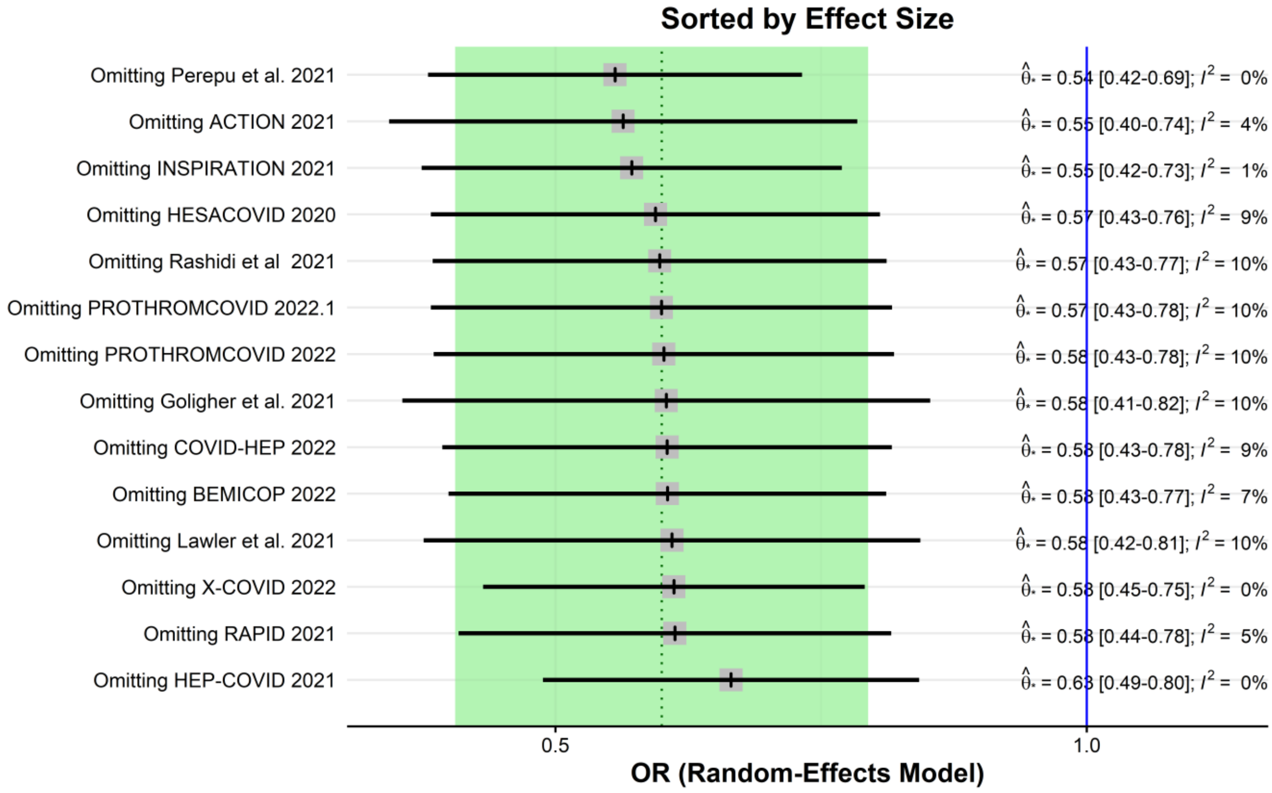

Figure S11: Major bleeding events in cohorts with different disease severity

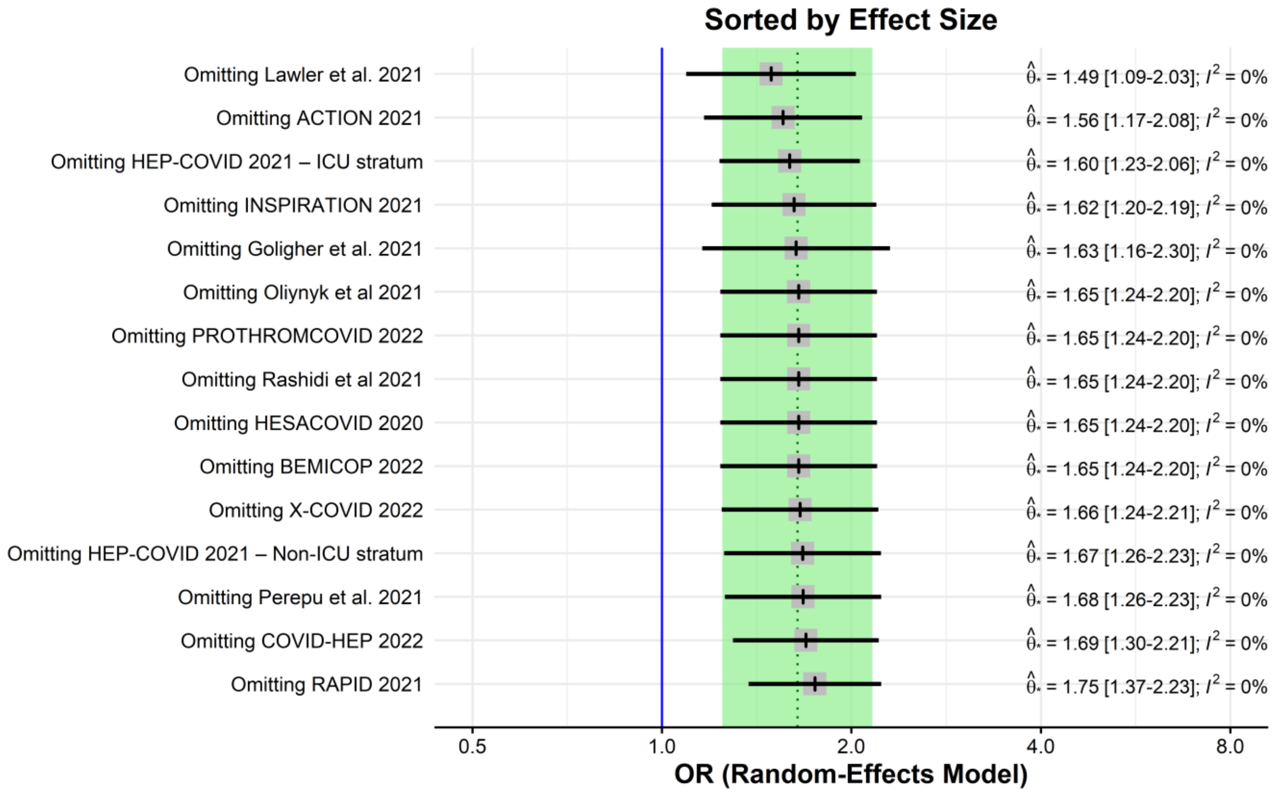

Figure S12: Major bleeding events in cohorts with different anticoagulant doses

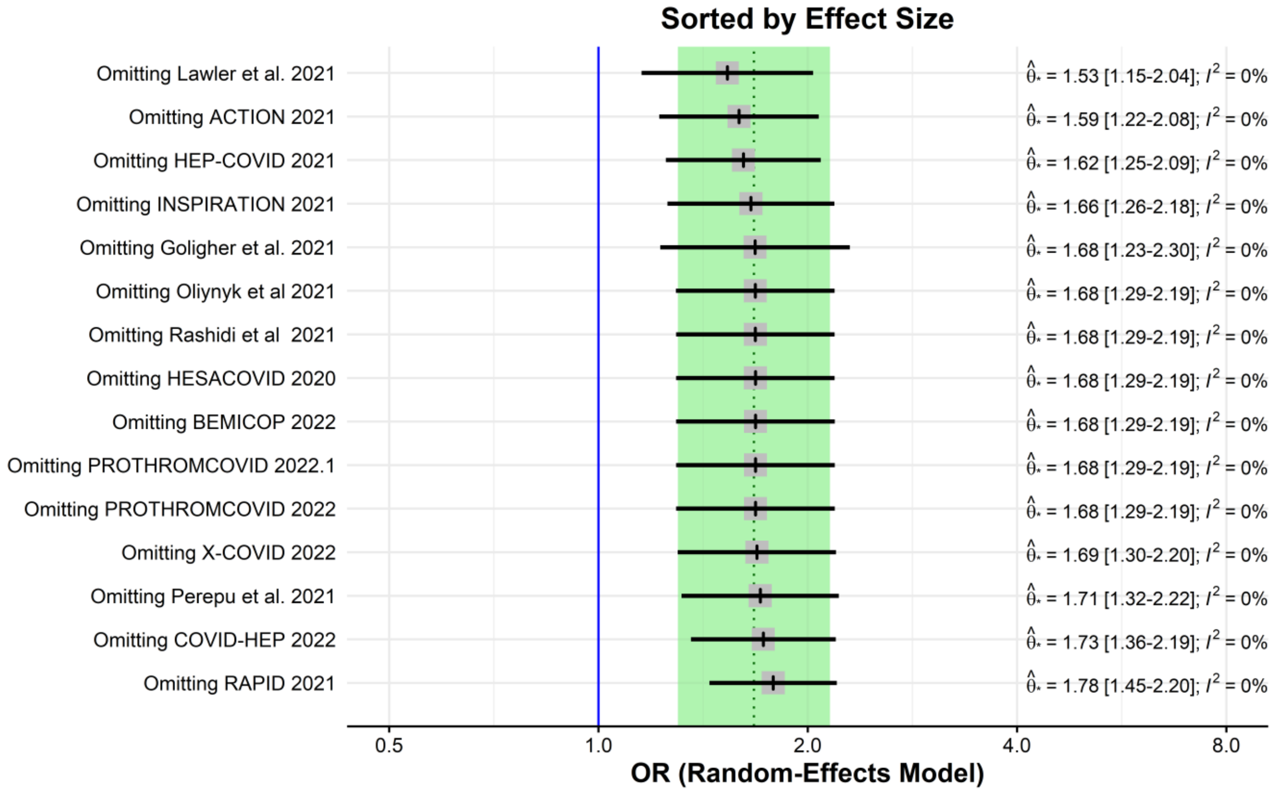

Figure S13: The need for invasive mechanical ventilation

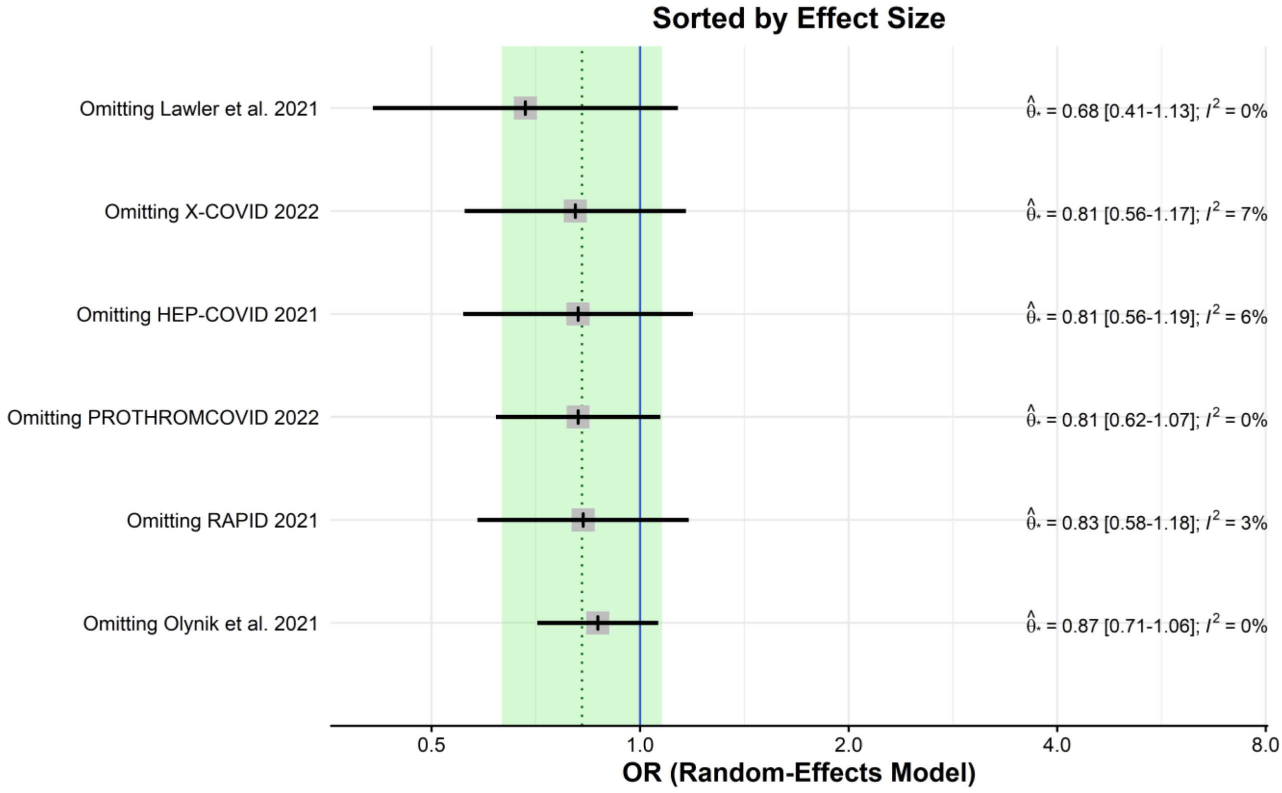

Excluded articles during full text selection (search on 18th October 2021)

tle	Author	Year	DOI	Eligibility	Reason for exclusion
[Anticoagulation in patients with COVID-19].	Pekrul, I	2021	DO - 10.1007/s00108-021-01190-y	Excluded	Commentary
Anticoagulation strategies and risk of bleeding events in critically ill COVID-19 patientsEstrategias de anticoagulación y riesgo de sangrado en pacientes ingresados en una Unidad de Cuidados Intensivos por COVID-19	Gabara, Cristina	2021	DO - 10.1016/j.medin.2021.07.004	Excluded	Review
[Not Available]	Fumeaux, Thierry	2021		Excluded	Comment on INSPIRATION study
Not Available]		2021	DO - 10.1055/a-1544-3994	Excluded	Video recording
A possible benefit from therapeutic anticoagulation in patients with coronavirus disease 2019: the Dolo hospital experience in Veneto, Italy.	Secco, Eleonora	2020	DO - 10.33963/KP.15489	Excluded	Observational study
Acute thromboembolic events (TE) within 30 days of COVID-19 infection in cancer patients	Zavras, P D	2021	DO - 10.1200/JCO.2021.39.15_suppl.e18691	Excluded	Meeting abstract
Anticoagulation and bleeding risk in patients with COVID-19.	Musoke, Nancy	2020	10.1016/j.thromres.2020.08.035	Excluded	Observational study
Anticoagulation Before Hospitalization Is a Potential Protective Factor for COVID-19: Insight From a French Multicenter Cohort Study.	Chocron, Richard	2021	DO - 10.1161/JAHA.120.018624	Excluded	Observational study
Anticoagulation in COVID-19: a single-center retrospective study.	Roomi, Sohaib Sanan	2021	10.1080/20009666.2020.1835297	Excluded	Observational study
Anticoagulation in COVID-19: Effect of Enoxaparin, Heparin, and Apixaban on Mortality.	Billett, H	2020	10.1055/s-0040-1720978	Excluded	Observational study
Anticoagulation management and outcomes in COVID-19 patients: a multi-center retrospective cohort study	Kaur, J	2020	10.1182/blood-2020-140997	Excluded	Observational study
Anticoagulation Therapy in Patients With Coronavirus Disease 2019: Results From a Multicenter International Prospective Registry (Health Outcome Predictive Evaluation for Corona Virus Disease 2019 [HOPE-COVID19]).	Santoro, Francesco	2021	10.1093/eurheartj/suaa198	Excluded	Observational study

Anticoagulation therapy using unfractionated heparin at a therapeutic dose for coronavirus disease 2019 patients with severe pneumonia: a retrospective historical control study.	Takayama, Wataru	2021	10.1002/ams2.679	Excluded	Observational study
ANTICOAGULATION, BLEEDING, AND IMMUNOTHROMBOSIS IN CRITICALLY ILL PATIENTS WITH COVID-19	Paez Vargas, J	2021	10.1016/j.chest.2021.07.926	Excluded	Observational study
Anticoagulation, Bleeding, Mortality, and Pathology in Hospitalized Patients With COVID-19.	Nadkarni, Girish N	2020	10.1016/j.jacc.2020.08.041	Excluded	Observational study
Association of anticoagulation dose and survival in hospitalized COVID-19 patients: A retrospective propensity score-weighted analysis.	Ionescu, Filip	2021	10.1111/ejh.13533	Excluded	Observational study
Association of Treatment Dose Anticoagulation With In-Hospital Survival Among Hospitalized Patients With COVID-19.	Paranjpe, Ishan	2020	10.1016/j.jacc.2020.05.001	Excluded	Observational study
Attention should be paid to venous thromboembolism prophylaxis in the management of COVID-19.	Wang, Tao	2020	10.1016/S2352-3026(20)30109-5	Excluded	Observational study
Beneficial Effects of Intermediate Dosage of Anticoagulation Treatment on the Prognosis of Hospitalized COVID-19 Patients: The ETHRA Study.	Poulakou, Garyphallia	2021	10.21873/invivo.12305	Excluded	Observational study
Bleeding prevalence in COVID-19 patients receiving intensive antithrombotic prophylaxis.	Kessler C.	2020	10.1007/s11239-020-02244-y	Excluded	Observational study
Bleeding risk by intensity of anticoagulation in critically ill patients with COVID-19: A retrospective cohort study.	Halaby, Rim	2021	10.1111/jth.15310	Excluded	Observational study
Bleeding risk in hospitalized patients with COVID-19 receiving intermediate- or therapeutic doses of thromboprophylaxis.	Demelo-Rodriguez, Pablo	2021	10.1111/jth.15400	Excluded	Observational study
Clinical and therapeutic outcomes of COVID-19 intensive care units (ICU) patients: a retrospective study in Ghana.	Afriyie-Mensah, Jane	2021	10.11604/pamj.2021.38.107.27131	Excluded	Observational study
Clinical Course and Risk Factors for In-Hospital Mortality of 205 Patients with	Turrini, Mauro	2021	10.3390/vaccines9060640	Excluded	Observational study

SARS-CoV-2 Pneumonia in Como, Lombardy Region, Italy.					
Clinical outcome with different doses of low-molecular-weight heparin in patients hospitalized for COVID-19.	Mennuni, Marco G	2021	10.1007/s11239-021-02401-x	Excluded	Observational study
Clinical Outcomes With the Use of Prophylactic Versus Therapeutic Anticoagulation in Coronavirus Disease 2019.	Motta, Jishu Kaul	2020	10.1097/CCE.0000000000000309	Excluded	Observational study
Combined anticoagulant and antiplatelet therapy is associated with an improved outcome in hospitalised patients with COVID-19: a propensity matched cohort study.	Matli, Kamal	2021	10.1136/openhrt-2021-001785	Excluded	Observational study
Comparison between Prophylactic versus Therapeutic Doses of Low-Molecular-Weight Heparin in Severely Ill Coronavirus Disease 2019 Patients in Relation to Disease Progression and Outcome	Elmelhat, A	2020	10.1159/000511163	Excluded	Observational study
Comparison of standard prophylactic and preemptive therapeutic low molecular weight heparin treatments in hospitalized patients with COVID-19.	Copur, B	2021	10.4149/BLL_2021_100	Excluded	Observational study
Consequences of impaired coagulation and impact of antithrombotic prophylaxys in elderly patients with coronavirus disease (COVID-19)	Asuncion, C G	2020	10.1007/s41999-020-00428-6	Excluded	Full text unavailable
Continuous Infusion Low-Dose Unfractionated Heparin for the Management of Hypercoagulability Associated With COVID-19	Li, M	2020	10.1177/0897190020966207	Excluded	Observational study
COVID-19 associated coagulopathy: Thrombosis, hemorrhage and mortality rates with an escalated-dose thromboprophylaxis strategy.	Daughety, Molly M	2020	10.1016/j.thromres.2020.10.004	Excluded	Observational study
Covid-19 thromboembolic complications and risk factors for poor outcomes: Experience from a single ICU	Bullman, L	2020	10.1186/s40635-020-00354-8	Excluded	Observational study
COVID-19-associated coagulopathy: safety and efficacy of prophylactic anticoagulation therapy in hospitalized adults with COVID-19	Chambers, I	2020	10.1182/blood-2020-141951	Excluded	Full text unavailable

COVID-19-RELATED COAGULOPATHY IN CRITICALLY ILL PATIENTS: EXPERIENCE FROM AN ACADEMIC MEDICAL CENTER	Thayyil Jayakrishnan, T	2021	10.1016/j.chest.2021.07.963	Excluded	Full text unavailable
COVID-19 Infection in Critically Ill Patients Carries a High Risk of Venous Thrombo-embolism.	Bellmunt-Montoya, Sergi	2021	10.1016/j.ejvs.2020.12.015	Excluded	Observational study
Deep vein thrombosis in noncritically ill patients with coronavirus disease 2019 pneumonia: deep vein thrombosis in non-intensive care unit patients.	Jimenez-Guiu, Xavier	2021	10.1016/j.jvsv.2020.08.028	Excluded	Observational study
Dosing of thromboprophylaxis and mortality in critically ill COVID-19 patients.	Jonmarker, Sandra	2020	10.1186/s13054-020-03375-7	Excluded	Observational study
Effect of Antithrombotic Therapy on Clinical Outcomes in Outpatients With Clinically Stable Symptomatic COVID-19: The ACTIV-4B Randomized Clinical Trial.	Connors, Jean M	2021	10.1001/jama.2021.17272	Included	
Effect of Intermediate-Dose vs Standard-Dose Prophylactic Anticoagulation on Thrombotic Events, Extracorporeal Membrane Oxygenation Treatment, or Mortality Among Patients With COVID-19 Admitted to the Intensive Care Unit: the INSPIRATION Randomized Clinic	Sadeghipour, P	2021	10.1001/jama.2021.4152	Included	
Effectiveness of therapeutic heparin versus prophylactic heparin on death, mechanical ventilation, or intensive care unit admission in moderately ill patients with covid-19 admitted to hospital: RAPID randomised clinical trial.	Sholzberg, Michelle	2021	10.1136/bmj.n2400	Included	
Efficacy and Safety of D-dimer, Weight, and Renal Function-Adjusted Thromboprophylaxis in Patients with Coronavirus Disease 2019 (COVID-19).	Arachchillage, Deepa R J	2021		Excluded	Observational study
Efficacy and Safety of Therapeutic-Dose Heparin vs Standard Prophylactic or Intermediate-Dose Heparins for Thromboprophylaxis in High-risk Hospitalized Patients With COVID-19: The HEP-COVID Randomized Clinical Trial.	Spyropoulos, Alex C	2021	10.1001/jamainternmed.2021.6203	Included	

Empiric Therapeutic Anticoagulation and Mortality in Critically Ill Patients With Respiratory Failure From SARS-CoV-2: A Retrospective Cohort Study.	Ferguson, John	2020	10.1002/jcph.1749	Excluded	Observational study
Empiric use of anticoagulation in hospitalized patients with COVID-19: a propensity score-matched study of risks and benefits.	Yu, Bo	2021	10.1186/s40364-021-00283-y	Excluded	Observational study
Enhanced thromboprophylaxis dosing or therapeutic anticoagulation are not associated with a decrease in thrombo-embolic events in patients with COVID19: A single centre experience	Karimi, A M	2020	10.1186/s40635-020-00354-8	Excluded	Full text unavailable
Evaluation of a patient specific, targeted-intensity pharmacologic thromboprophylaxis protocol in hospitalized patients with COVID-19.	Farrar, Julie E	2021	10.1007/s11239-021-02552-x	Excluded	Observational study
EVALUATION OF THROMBOEMBOLIC EVENTS AND ANTICOAGULANT STRATEGIES IN PATIENTS WITH COVID-19: A RETROSPECTIVE ANALYSIS	Lyn Dela Cruz, J	2021	10.1016/j.chest.2021.07.1024	Excluded	Full text unavailable
Hemorrhagic stroke and anticoagulation in COVID-19.	Dogra, Sidhant	2020	10.1016/j.jstrokecerebrovasdis.2020.104984	Excluded	Observational study
Hemorrhagic stroke in the setting of COVID-19 is associated with anticoagulation use	Kvernland, A	2021	10.1161/str.52.suppl-1.P100	Excluded	Full text unavailable
Heparin in COVID-19 Patients Is Associated with Reduced In-Hospital Mortality: the Multicenter Italian CORIST Study	Di Castelnuovo	2021	DOI: 10.1055/a-1347-6070	Excluded	Observational study
Heparin for Moderately Ill Patients with Covid-19.	Sholzberg, Michelle	2021	10.1101/2021.07.08.21259351	Included	RAPID trial - duplicate
High incidence of venous thromboembolic events in anticoagulated severe COVID-19 patients.	Llitjos, Jean-François	2020	10.1111/jth.14869	Excluded	Observational study
High risk of thrombosis in patients with severe SARS-CoV-2 infection: a multicenter prospective cohort study	helms J.	2020	10.1007/s00134-020-06062-x	Excluded	Observational study
Higher anticoagulation targets and risk of thrombotic events in severe COVID-19 patients: bi-center cohort study.	Helms, Julie	2021	10.1186/s13613-021-00809-5	Excluded	Observational study

Higher heparin dosages reduce thromboembolic complications in patients with COVID-19 pneumonia.	Carallo, Claudio	2021	10.1136/jim-2020-001628	Excluded	Observational study
Higher Intensity Thromboprophylaxis Regimens and Pulmonary Embolism in Critically Ill Coronavirus Disease 2019 Patients.	Taccone, Fabio Silvio	2020	10.1097/CCM.0000000000004548	Excluded	Observational study
Hospital mortality and safety of therapeutic vs. prophylactic doses of low molecular weight heparin in COVID-19 patients	Aschieri, D	2020	10.1093/eurheartj/suaa198	Excluded	Full text unavailable
IMPACT OF FULL DOSE ANTICOAGULATION IN HOSPITALIZED PREDOMINANTLY BLACK PATIENTS WITH SARS-COV-2 INFECTION: a RETROSPECTIVE SINGLE CENTER EXPERIENCE	Samaha G.	2020	10.1016/j.chest.2020.09.017	Excluded	Full text unavailable
Impact of High-Dose Prophylactic Anticoagulation in Critically Ill Patients With COVID-19 Pneumonia.	Tacquard, Charles	2021	10.1016/j.chest.2021.01.017	Excluded	Observational study
IMPACT OF THROMBOPROPHYLACTIC STRATEGY ON BLEEDING RISK AMONG IN- AND OUTPATIENTS WITH COVID-19	Svet M	2021	10.1016/S0735-1097(21)04466-1	Excluded	Full text unavailable
Impact of treatment and anticoagulation on thrombosis in COVID-19 patients	Warrior, S	2020	10.1182/blood-2020-137707	Excluded	Full text unavailable
In COVID-19, therapeutic vs. prophylactic anticoagulation did not improve clinical outcomes and increased bleeding.	Diep, Robert	2021	10.7326/ACPJ202110190-112	Excluded	Full text unavailable
Incidence and mortality of thrombotic complications ICU patients with COVID-19	Llorente Ruiz, B	2020	10.1186/s40635-020-00354-8	Excluded	Full text unavailable
Incidence and risk factors associated with bleeding complications in COVID patients	Acha Aranda, A	2020	10.1186/s40635-020-00354-8	Excluded	Full text unavailable
Incidence of deep vein thrombosis through an ultrasound surveillance protocol in patients with COVID-19 pneumonia in non-ICU setting: A multicenter prospective study.	Pieralli, Filippo	2021	10.1371/journal.pone.0251966	Excluded	Observational study
Incidence of Venous Thromboembolism and Effect of Anticoagulant Dosing in Hospitalized COVID-19 Patients.	Kumar, Gagan	2020	10.14740/jh836	Excluded	Observational study

Incidence of Venous Thromboembolism in Critically Ill Coronavirus Disease 2019 Patients Receiving Prophylactic Anticoagulation.	Trigonis, Russell A	2020	10.1097/CCM.0000000000004472	Excluded	Observational study
Incidence of venous thromboembolism in hospitalized patients with COVID-19.	Middeldorp, Saskia	2020	10.1111/jth.14888	Excluded	Observational study
Increased anticoagulation reduces proximal deep vein thrombosis in mechanically ventilated COVID-19 patients: Venous thrombosis prevention & COVID-19.	Voicu, Sebastian	2021	10.1016/j.jinf.2020.11.019	Excluded	Observational study
Increasing dosages of low-molecular-weight heparin in hospitalized patients with Covid-19.	Martinelli, I	2021	10.1007/s11739-020-02585-9	Excluded	Observational study
Increasing doses of anticoagulation are associated with improved survival in hospitalized COVID-19 patients	Ionescu, F	2020	10.1182/blood-2020-134835	Excluded	Full text unavailable
Intensity of anticoagulation and survival in patients hospitalized with COVID-19 pneumonia.	Hsu, Andrew	2020	10.1016/j.thromres.2020.09.030	Excluded	Observational study
Intermediate versus standard dose heparin prophylaxis in COVID-19 ICU patients: A propensity score-matched analysis.	Moll, Matthew	2021	10.1016/j.thromres.2021.04.009	Excluded	Observational study
Intermediate vs Standard-dose Prophylactic Anticoagulation in Patients with COVID-19 Admitted to ICU: ninety-day Results from the INSPIRATION Trial		2021	10.1055/a-1485-2372	Included	Same study pool, another outcome
Intermediate-dose anticoagulation, aspirin, and in-hospital mortality in COVID-19: a propensity score-matched analysis.	Meizlish, Matthew L	2021	10.1101/2021.01.12.21249577	Excluded	Observational study
Is there a possible benefit in therapeutic anticoagulation for all COVID-19 patients?	Aly, H	2020	10.1186/s40635-020-00354-8	Excluded	Full text unavailable
Low incidence of thrombotic events in SARS-CoV-2 mechanically ventilated anticoagulated patients	Perot, A	2020	10.1186/s40635-020-00354-8	Excluded	Full text unavailable
Low molecular weight heparin and 28-day mortality among patients with coronavirus disease 2019: A cohort study in the early epidemic era.	Qin, Wei	2021	10.1016/j.thromres.2020.11.020	Excluded	Observational study
Low molecular weight heparin at high dose impacts on outcome of COVID-19	Bonetti, S	2021	10.1097/01.hjh.0000746768.43077.0d	Excluded	Full text unavailable

hospitalized patients, while standard dose doesn't					
Low molecular weight heparin is useful in adult COVID-19 inpatients. Experience during the first Spanish wave: observational study.	Gonzalez-Porras, Jose Ramon	2021	10.1590/1516-3180.2021.0098.R1.08062021	Excluded	Observational study
Low Rate of Thrombosis in Mexican Patients with COVID-19 Infection. a Benefit of Higher Doses Anticoagulants or a Sub Diagnosis?	Marquez, F G	2020	10.1182/blood-2020-136240	Excluded	Full text unavailable
Low-molecular-weight heparin dosing and anti-xa level monitoring in critically ill COVID-19 adults	Mioni, R	2021	10.1097/01.ccm.0000727112.58380.5e	Excluded	Full text unavailable
Major bleeding complications in critically ill patients with COVID-19 pneumonia.	Godier, Anne	2021	10.1007/s11239-021-02403-9	Excluded	Observational study
Management of heparin in COVID-19 patients: A single-centre experience	Sottilotta, G	2020	10.2450/2020.S4	Excluded	Full text unavailable
Missed cerebrovascular events during prolonged sedation for COVID-19 pneumonia.	Bruce, Samuel S	2021	10.1016/j.jocn.2021.01.008	Excluded	Observational study
Mortality and transfusion requirements in COVID-19 hospitalized italian patients according to severity of the disease	Grandone, E	2021	10.3390/jcm10020242	Excluded	Observational study
Outcomes in COVID-19 patients on treatment dose anti- coagulation compared to prophylactic dose anti-coagulation	Kodama, R	2020	10.1182/blood-2020-142552	Excluded	Observational study
Pattern of anticoagulation prescription for patients with Covid-19 acute respiratory distress syndrome admitted to ICU. Does it impact outcome?	Nadeem, Rashid	2021	10.1016/j.hrtlng.2020.10.009	Excluded	Observational study
Predictors of acute deep venous thrombosis in patients hospitalized for COVID-19.	Riyahi, Sadjad	2021	10.1097/MD.0000000000027216	Excluded	Observational study
Preliminary Experience With Low Molecular Weight Heparin Strategy in COVID-19 Patients.	Paolisso, Pasquale	2020	10.3389/fphar.2020.01124	Excluded	Observational study
Prevalence and Predictors of Venous Thromboembolism or Mortality in Hospitalized COVID-19 Patients.	Cohen, Stuart L	2021	10.1055/a-1366-9656	Excluded	Observational study
Prevalence of deep vein thrombosis in Italian Covid-19 hospitalized patients	Baccellieri, D	2020	10.2450/2020.S4	Excluded	Full text unavailable

Prevalence, predictors and outcomes of bleeding events in patients with COVID-19 infection on anticoagulation: Retrospective cohort study.	Alkhamis, Ahmed	2021	10.1016/j.amsu.2021.102567	Excluded	Observational study
Preventing Thrombohemorrhagic Complications of Heparinized COVID-19 Patients Using Adjunctive Thromboelastography: A Retrospective Study.	Bunch, Connor M	2021	10.3390/jcm10143097	Excluded	Observational study
Prognostic factors and predictors of outcome in patients with COVID-19 and related pneumonia: a retrospective cohort study.	Boari, Gianluca E M	2020	10.1042/BSR20203455	Excluded	Observational study
Prophylactic or therapeutic doses of heparins for COVID-19 infection? A retrospective study.	Bolzetta, Francesco	2021	10.1007/s40520-020-01750-6	Excluded	Observational study
Prophylactic versus therapeutic anticoagulation for survival of patients with COVID-19 on steroid.	Kuno, Toshiki	2021	10.1007/s11239-021-02569-2	Excluded	Observational study
Pulmonary embolism in patients with severe COVID-19 treated with intermediate- to full-dose enoxaparin: A retrospective study.	Scarduelli, Cleante	2021	10.4081/monaldi.2021.1758	Excluded	Observational study
Risk Factors for Mortality from COVID-19 in a New York Inner city Epicenter	Majzoub, M	2021	10.1016/j.jaci.2020.12.295	Excluded	Observational study
Risk factors for systemic and venous thromboembolism, mortality and bleeding risks in 1125 patients with COVID-19: relationship with anticoagulation status.	Li, Wencheng	2021	10.18632/aging.202769	Excluded	Observational study
Real world use of anticoagulation among hospitalized patients with COVID-19 in the United States	Bates, B	2021	10.1002/pds.5305	Excluded	Observational study
Real-time collection of data on patients with COVID-19 and thrombosis and/or haemorrhage has aided the development of thromboembolism prophylaxis guidelines at the Royal Surrey County Hospital during the COVID-19 pandemic.	Simpson G.	2021	10.7861/clinmed.21-2-s51	Excluded	Observational study
Reduction of venous thromboembolic events in hospitalized patients with coronavirus disease 2019 after intensification of thromboprophylaxis	Filippidis	2020	10.1002/rth2.12413	Excluded	Full text unavailable

ROLE OF THROMBODYNAMICS GLOBAL COAGULATION TEST IN IMPROVING TREATMENT RESULTS IN PATIENTS WITH CORONAVIRUS INFECTION AT A COVID-19 HOSPITAL.	Krylov A.	2021		Excluded	Full text unavailable
Safety and efficacy of anticoagulation in critically ill patients with COVID-19	Shuler, E	2021	10.1164/ajrccm-conference.2021.203.1_MeetingAbstracts.A2647	Excluded	Full text unavailable
Safety profile of enhanced thromboprophylaxis strategies for critically ill COVID-19 patients during the first wave of the pandemic: observational report from 28 European intensive care units.	Lavinio, Andrea	2021	10.1186/s13054-021-03543-3	Excluded	Observational study
Standard prophylactic versus intermediate dose enoxaparin in adults with severe COVID-19: A multi-center, open-label, randomized controlled trial.	Perepu Usha S	2021	10.1111/jth.15450	Included	
STANDARD VS HIGH-INTENSITY PROPHYLACTIC ANTICOAGULATION IN COVID-19: A US, CALIFORNIAN RETROSPECTIVE STUDY	Almadi T		10.1016/j.chest.2021.07.1305	Excluded	Full text unavailable
The effect of anticoagulation on clinical outcomes in novel Coronavirus (COVID-19) pneumonia in a U.S. cohort.	Lynn, Lei	2021	10.1016/j.thromres.2020.10.031	Excluded	Observational study
The effect of anticoagulation on mortality in COVID-19 patients: The drug, the dose, and the D-Dimer	Bilett H	2020	10.1002/rth2.12413	Excluded	Full text unavailable
The hazard of (sub)therapeutic doses of anticoagulants in non-critically ill patients with Covid-19: The Padua province experience.	Pesavento, Raffaele	2020	10.1111/jth.15022	Excluded	Observational study
The Impact of Different Prophylactic Anticoagulation Doses on the Outcomes of Patients with COVID-19	Rodolfo	2021	10.1016/j.thromres.2021.02.031	Excluded	Observational study
The impact of protocol-based high-intensity pharmacological thromboprophylaxis on thrombotic events in critically ill COVID-19 patients.	Atallah, B	2021	10.1111/anae.15300	Excluded	Observational study
The Impact of Risk-Adjusted Heparin Regimens on the Outcome of Patients with	Di Micco, Pierpaolo	2021	10.3390/v13091720	Excluded	Observational study

COVID-19 Infection. A Prospective Co-hort Study.					
The original and modified Caprini score equally predicts venous thromboembolism in COVID-19 patients.	Tsaplin, S	2021	10.1016/j.jvsv.2021.02.018	Excluded	Observational study
The Potential Role of Therapeutic Dose of Low Molecular Weight Heparin (LWMH) to Attenuate Hyper-Inflammatory State in Hospitalized COVID-19 Patients	Mughal M S	2020	10.1182/blood-2020-142109	Excluded	Full text unavailable
The role of anticoagulation therapy in management of COVID-19 patients	Shahid S	2021	10.1136/thorax-2020-BTSabstracts.104	Excluded	Full text unavailable
The role of D-dimer for optimal thrombo-prophylaxis strategy in patients with COVID-19	Bhoopat L	2020	10.1182/blood-2020-141732	Excluded	Observational study
Therapeutic Anticoagulation Delays Death in COVID-19 Patients: Cross-Sectional Analysis of a Prospective Cohort.	Ionescu, Filip	2020	10.1055/s-0040-1716721	Excluded	Observational study
Therapeutic Anticoagulation with Heparin in Critically Ill Patients with Covid-19.	Goligher, Ewan C	2021	10.1056/NEJMoa2103417	Included	
Therapeutic Anticoagulation with Heparin in Noncritically Ill Patients with Covid-19.	Lawler, Patrick R	2021	10.1056/NEJMoa2105911	Included	
Therapeutic dosing of low-molecular-weight heparin may decrease mortality in patients with severe COVID-19 infection.	Canoglu Kadir	2020	10.5144/0256-4947.2020.462	Excluded	Observational study
Therapeutic versus prophylactic anticoag-ulation for patients admitted to hospital with COVID-19 and elevated D-dimer concentration (ACTION): an open-label, multicentre, randomised, controlled trial.	Lopes, Renato D	2021	10.1016/S0140-6736(21)01203-4	Included	
Therapeutic versus prophylactic anticoag-ulation for severe COVID-19: A random-ized phase II clinical trial (HESACOVID).	Lemos, Anna Cristina Ber-toldi	2020	10.1016/j.throm-res.2020.09.026	Included	
Therapeutic vs. prophylactic bemiparin in hospitalized patients with non-severe COVID-19 (BEMICOP): an open-label, multicenter, randomized trial.	Marcos, María	2021	10.1055/a-1667-7534	Included	
Thrombo-embolic events associated with Covid-19 ARDS - Epidemiology and risk factors	De Mont-mollin, E	2020	10.1186/s40635-020-00354-8	Excluded	Full text unavailable
Thromboinflammatory state and venous thromboembolic events in patients with		2021	10.20452/pamw.15625	Excluded	Observational study

coronavirus disease 2019 admitted to a nonintensive care unit: A prospective study					
Thromboprophylaxis in Critically Ill Coronavirus Disease 2019 Patients.	Piagnerelli, Michaël	2020	10.1097/CCE.0000000000000177	Excluded	Observational study
Thromboprophylaxis and the D-Dimer in critically unwell patients with Covid-19. A UK single centre experience	Tsiamita, O	2021	10.1111/bjh.17492	Excluded	Full text unavailable
Thromboses and COVID-19: reducing inflammation in addition to thromboprophylaxis.	Mehta, Puja	2021	10.1016/S2665-9913(21)00003-5	Excluded	Observational study
Thrombosis and anticoagulation in COVID-19.	Ramos, Roberta Pulcheri	2020	10.36416/1806-3756/e20200317	Excluded	Observational study
Thrombotic complications and anticoagulation in COVID-19 pneumonia: a New York City hospital experience.	Hanif, Ahmad	2021	10.1007/s00277-020-04216-x	Excluded	Observational study
Thrombotic Complications in a Canadian Population of Critically Ill Patients with COVID-19	Camille, S	2020	10.1182/blood-2020-141996	Excluded	Observational study
Trends in Venous Thromboembolism Anticoagulation in Patients Hospitalized With COVID-19.	Vaughn, Valerie M	2021	10.1001/jamanetworkopen.2021.11788	Excluded	Observational study
Utility of D-dimers and intermediate-dose prophylaxis for venous thromboembolism in critically ill patients with COVID-19.	Zermatten, Maxime G	2020	10.1016/j.thromres.2020.08.027	Excluded	Observational study
Venous and arterial thromboembolic complications in COVID-19 patients admitted to an academic hospital in Milan, Italy.	Lodigiani, Corrado	2020	10.1016/j.thromres.2020.04.024	Excluded	Observational study
Venous Thrombo-Embolism in Hospitalized SARS-CoV-2 Patients Treated with Three Different Anticoagulation Protocols: Prospective Observational Study.	Longhitano, Yaroslava	2020	https://doi.org/10.3390/biology9100310	Excluded	Observational study
Venous thromboembolism and bleeding in critically ill COVID-19 patients treated with higher than standard low molecular weight heparin doses and aspirin: A call to action.	Pavoni, Vittorio	2020	10.1016/j.thromres.2020.09.013	Excluded	Observational study
VTE PROPHYLAXIS STRATEGIES WITH ENOXAPARIN DURING THE COVID-19 PANDEMIC	Nguyen, Huan Mark	2021	10.1016/j.chest.2021.07.1299	Excluded	Full text unavailable

Excluded articles during full text selection (search on 23rd May 2022)

Title	Author	Year	DOI	Eligibility	Reason for exclusion
Comparison of the Effect of Unfractionated Heparin and Enoxaparin Sodium at Different Doses on the Course of COVID-19-Associated Coagulopathy	Olyinyk, Oleksandr	2021	.https://doi.org/10.3390/life11101032	Included	
Effect of Antithrombotic Therapy on Clinical Outcomes in Outpatients With Clinically Stable Symptomatic COVID-19: The ACTIV-4B Randomized Clinical Trial.	Connors, Jean M	2021	10.1001/jama.2021.17272	Included	Already included in previous search
Efficacy and safety of fondaparinux versus unfractionated heparin in patients hospitalised with severe COVID-19 pneumonia and coagulopathy: a randomised, open-label clinical trial	Adrian, L H	2022	10.1093/eurheartj/ehab849.169	Excluded	Full text unavailable
Effectiveness of therapeutic heparin versus prophylactic heparin on death, mechanical ventilation, or intensive care unit admission in moderately ill patients with covid-19 admitted to hospital: RAPID randomised clinical trial.	Sholzberg, Michelle	2021	10.1136/bmj.n2400	Included	Already included in previous search
Enoxaparin for thromboprophylaxis in hospitalized COVID-19 patients: the X-COVID-19 Randomized Trial	Morici, N	2022	10.1111/eci.13735	Included	
High-Dose Prophylactic Anticoagulation in Severe COVID-19 Pneumonia.	Nadeem, Rashid	2021	10.1055/a-1485-2372	Excluded	Observational study
Higher-dose versus standard-dose prophylactic anticoagulation in hospitalized patients with	Long, Brit	2022	10.1111/acem.14417	Excluded	Review

COVID-19.					
Intermediate-Dose versus Standard-Dose Prophylactic Anticoagulation in Patients with COVID-19 Admitted to the Intensive Care Unit: 90-Day Results from the INSPIRATION Randomized Trial.	Bikdeli, Behnood	2021	10.1055/a-1485-2372	Included	Already included in previous search
Optimal Patient Selection for the Prophylactic Anticoagulation Therapy in Patients With Coronavirus Disease 2019.	Nishioka, Ayane	2021	10.1097/CCM.0000000000005158	Excluded	Commentary
Optimal thromboprophylaxis strategies in non-critically ill patients with COVID-19 pneumonia. The PROTHROMCOVID Randomized Controlled Trial	Muñoz-Rivas, Nuria	2022	10.1101/2022.05.03.22274594	Included	
Oral Rivaroxaban in the Prophylaxis of COVID-19 Induced Coagulopathy.	Kumar, Dhiraj	2022	PMID: 35436816	Excluded	Full text unavailable
Oral rivaroxaban versus subcutaneous enoxaparin in the prophylaxis of covid-19 induced coagulopathy in mild to moderate sars cov-2 infection	Km, V	2021	10.1161/circ.144.suppl-1.10011	Excluded	Full text unavailable
Standard prophylactic versus intermediate dose enoxaparin in adults with severe COVID-19: A multi-center, open-label, randomized controlled	Perepu, Usha	2021	10.1111/jth.15450	Included	Already included in previous search

trial.					
The role of heparin in reducing in-hospital complications and three-month mortality rates in hospitalized COVID-19 patients.	Calabretta, F	2022	10.1016/j.ejim.2022.03.015	Excluded	Observational study
Therapeutic anticoagulation in COVID-19 patients.	Belfaqeeh, O	2021	10.1016/j.thromres.2021.02.006	Excluded	Commentary
Therapeutic Anticoagulation with Heparin in Critically Ill Patients with Covid-19.	Goligher, Ewan C	2021	10.1056/NEJMoa2103417	Included	Already included in previous search
Therapeutic Anticoagulation with Heparin in Noncritically Ill Patients with Covid-19.	Lawler, Patrick R	2021	10.1056/NEJMoa2105911	Included	Already included in previous search
Therapeutic anticoagulation to prevent thrombosis, coagulopathy, and mortality in severe COVID-19: The Swiss COVID-HEP randomized clinical trial.	Blondon, Marc	2022	10.1002/rth2.12712	Included	
Therapeutic versus prophylactic anticoagulation for patients admitted to hospital with COVID-19 and elevated D-dimer concentration (ACTION): an open-label, multicentre, randomised, controlled trial.	Lopes, Renato D	2021	10.1016/S0140-6736(21)01203-4	Included	Already included in previous search
Therapeutic versus Prophylactic Bemiparin in Hospitalized Patients with Nonsevere COVID-19 Pneumonia (BEMICOP Study): An Open-Label, Multicenter, Randomized, Controlled Trial	Marcos-Jubilar, M	2022	10.1055/a-1667-7534	Included	Already included in previous search
Thrombosis and bleeding in critically ill COVID-19 patients at higher than prophylactic dose of heparins	Mavri, A	2021	10.1002/rth2.12589	Excluded	Full text unavailable
Tissue plasminogen activator for the treatment of adults with critical COVID-19: A pilot randomized clinical trial	Rashidi, Fahrid	2021	https://doi. org/10.1016/j.thromres.2021.12 .003	Included	
